# Identification of antigen-presenting cell-T cell interactions driving immune responses to food

**DOI:** 10.1126/science.ado5088

**Published:** 2025-03-14

**Authors:** Maria C.C. Canesso, Tiago B.R. Castro, Sandra Nakandakari-Higa, Ainsley Lockhart, Julia Luehr, Juliana Bortolatto, Roham Parsa, Daria Esterházy, Mengze Lyu, Tian-Tian Liu, Kenneth M. Murphy, Gregory F. Sonnenberg, Bernardo S. Reis, Gabriel D. Victora, Daniel Mucida

**Affiliations:** 1 Laboratory of Mucosal Immunology, The Rockefeller University, New York, United States; 2 Laboratory of Lymphocyte Dynamics, The Rockefeller University, New York, United States; 3 Department of Pathology, University of Chicago, Chicago, United States; 4 Joan and Sanford I. Weill Department of Medicine, Division of Gastroenterology & Hepatology, Department of Microbiology and Immunology, Jill Roberts Institute for Research in Inflammatory Bowel Disease, Weill Cornell Medicine, Cornell University, New York, United States; 5 Department of Pathology and Immunology, Washington University in St Louis, School of Medicine, St Louis, United States; 6 Howard Hughes Medical Institute, The Rockefeller University, New York, United States

## Abstract

The intestinal immune system must concomitantly tolerate food and commensals and protect against pathogens. Antigen-presenting cells (APCs) orchestrate these immune responses by presenting luminal antigens to CD4^+^ T cells and inducing their differentiation into regulatory (pTreg) or inflammatory (Th) subsets. We used a proximity labelling method (LIPSTIC) to identify APCs that presented dietary antigens under tolerizing and inflammatory conditions and understand cellular mechanisms by which tolerance to food is induced and can be disrupted by infection. Helminth infections disrupted tolerance induction proportionally to the reduction in the ratio between tolerogenic APCs, including migratory dendritic cells (cDC1s) and Rorγt^+^ APCs, and inflammatory APCs, that were primarily cDC2s. These inflammatory cDC2s expanded by helminth infection did not present dietary antigens, thus avoiding diet-specific Th2 responses.

## One Sentence Summary

Ratio of inflammatory to tolerogenic APCs constrains differentiation of regulatory T cells and tolerance towards dietary antigens.

## Introduction

The balance between pro-inflammatory and tolerogenic immune responses depends on interactions between antigen presenting cells (APCs) and T cells (1, 2). In the intestine, APCs, particularly dendritic cells (DCs), take up soluble antigens, such as dietary and microbial proteins, from the intestinal lumen and then migrate to the gut-draining lymph nodes (gLNs), where they present these antigens to naïve T cells (3). This is complemented by presentation of soluble antigen that drains through lymphatics and is taken up by gLN-resident APCs (4). By inducing differentiation of regulatory or pro-inflammatory CD4^+^ T cell functional subsets during antigen presentation, these APCs determine the type of immune response that is directed towards each individual intestinal antigen (5–8). Although intestinal APC subsets with tolerogenic and immunogenic functions have been described (9–15), it remains unclear how APC-T cell interactions are organized to enable these distinct immune responses to take place simultaneously within the same lymphoid organs. Tolerance to food is dependent on the induction of peripheral regulatory T cells (pTregs) specific for dietary antigens (16). Both classical DCs subsets, cDC1 (CD103^+^ CD11b^−^) and cDC2 (CD103^+/−^ CD11b^+^), have been implicated in the induction of food-specific Foxp3^+^ pTregs (6, 17–19). More recently, Rorγt^+^ APCs, including ILC3s, Janus cells (JCs) or Thetis cells (TCs), were shown to be involved on microbiota-derived antigen presentation and induction of Foxp3^+^Rorγt^+^ Tregs (13–15). Overall, studies using traditional gene deletion strategies suffer from issues of specificity and/or poor targeting efficiency, as well as from compensatory effects whereby the task of a genetically-ablated APC subset is taken on by an alternative population (1). Therefore, conclusively determining how APCs dictate tolerance or inflammation in the gut would require the ability to identify specific APC subsets that present antigens to T cells in unmanipulated settings. To this end, we used Labelling Immune Partnerships by SorTagging Intercellular Contacts (LIPSTIC), a proximity-dependent labeling technology, that allows for identification of APCs engaged in presentation of a given antigen *in vivo* (20, 21).

## Results

### LIPSTIC identifies APCs presenting dietary antigens in the gLNs

**Figure 1.**
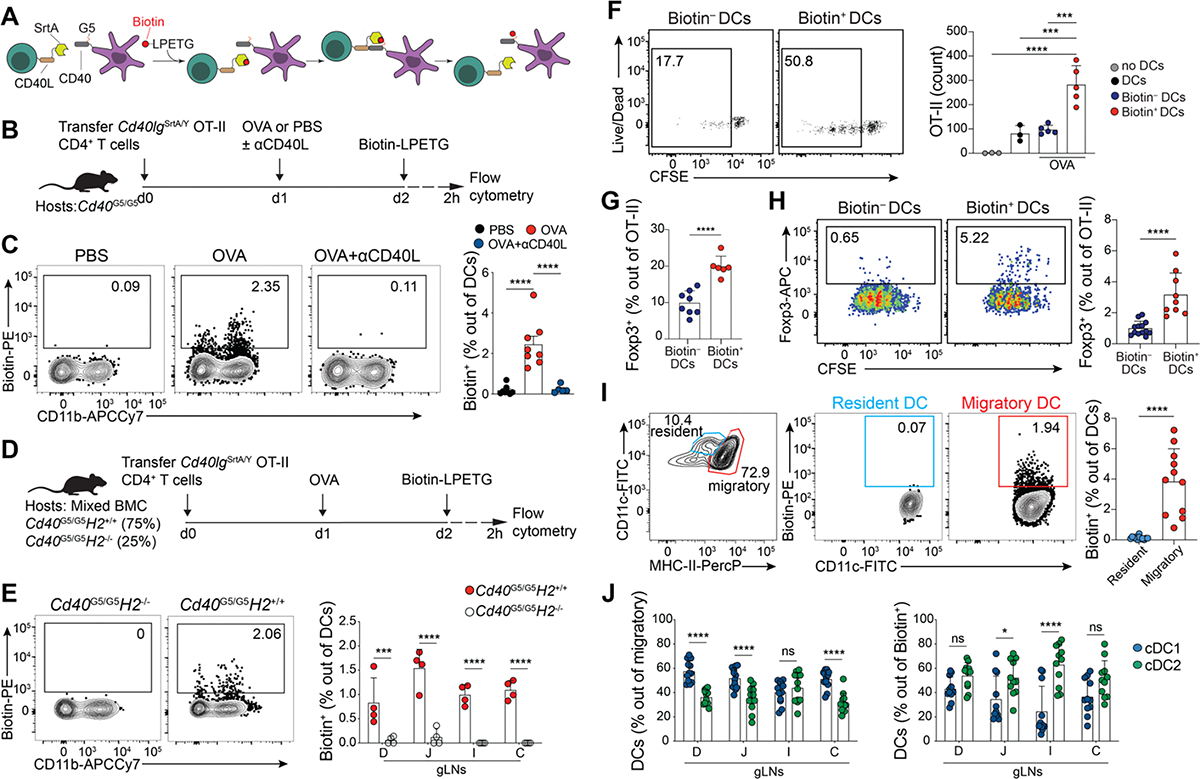
Using LIPSTIC to identify DCs presenting dietary antigens in the gLNs. (A) Schematic representation of LIPSTIC labeling of intercellular contacts *in vivo*. **(B, C, F-J)** CD45.2 *Cd40*^G5/G5^ mice were adoptively transferred with 1 × 10^6^ naive CD45.1 CD4^+^ *Cd40lg*^SrtA/Y^ OT-II T cells prior to one dose of intragastric PBS, OVA or OVA + anti CD40L antibody. Cell-cell interaction was revealed by LIPSTIC protocol 24 h later. **(B)** Experimental setup for panel *c*. **(C)** Representative flow plots showing percentage of labeled DCs in the duodenal gLNs (D-gLNs) (left) and quantification of data (right) (n = 3, 4 mice per group, pool of two independent experiments). **(D, E)** Mixed bone marrow chimera (BMC) mice reconstituted with *Cd40*^G5/G5^ and *Cd40*^G5/G5^*:H2*^*−/−*^ cells. Mice were adoptively transferred with 1 × 10^6^ naïve CD4^+^ *Cd40lg*^SrtA/Y^ OT-II T cells prior to one dose of intragastric OVA. Cell-cell interaction was revealed by LIPSTIC protocol 24 h later. **(D)** Experimental setup for panel (E). **(E)** Representative flow plots showing percentage of labeled DCs (n = 4 mice per group, representative of two independent experiments). **(F, G)** Sorted D-gLNs biotin^−^ or biotin^+^ DCs or DCs derived from OVA-naive mice were co-culture *in vitro* with naïve OT-II CFSE-labeled T cells for 96 h prior analysis. **(F)** Representative flow plots showing proliferation of OT-II T cells co-cultured with biotin^−^ or biotin^+^ DCs (left) and quantification of OT-II T cells per well at the end of the culture period with indicated DCs (right). **(G)** Percentage of Foxp3^+^ cells among proliferated OT-II T cells co-culture with biotin^−^ or biotin^+^ DCs. Each dot represents one mouse (n = 3 to 5 mice per group, representative of two independent experiments). **(H)** Representative flow plots showing percentage of Foxp3^+^ cells among proliferated OT-II T cells co-culture with biotin^−^ or biotin^+^ DCs in the presence of exogenous OT-II peptide (left), and quantification of data (right). Each dot represents one mouse (n = 3, 4 mice per group, pool of three independent experiments). **(I, J)** CD45.2 *Cd40*^G5/G5^ mice that were adoptively transferred with 1 × 10^6^ naive CD45.1 CD4^+^ *Cd40lg*^SrtA/Y^ OT-II T cells prior to one dose of intragastric OVA. Analyses were carried out 24 h later. (**I**) Representative flow plots showing gating on resident and migratory DCs in D-gLNs (left), percentage of labeled resident and migratory DCs in D-gLNs (center), and quantification of data (right). Each dot represents one mouse (n = 3 mice per group, pool of three independent experiments). **(J)** Percentage of cDC1 and cDC2 out of total migratory DCs (left) and percentage of cDC1 and cDC2 out of biotin^+^ DCs (right). Each dot represents one mouse (n = 3 mice per group, pool of three independent experiments). cDC1s were defined as Aqua^−^ CD45.2^+^ CD45.1^−^ Lin^−^CD11c^hi^ MHC-II^hi^ CD103^+^ CD11b^−^ and cDC2s were defined as Aqua^−^ CD45.2^+^ CD45.1^−^ Lin^−^ CD11c^hi^MHC-II^hi^ CD103^+/−^ CD11b^−^. D, duodenum; J, jejunum; I, ileum; C, colon. In graphs, the height of bars indicate mean, and error bars indicate SD. *P*-values were calculated by one-way ANOVA in (C), two-way ANOVA in (E), (F), and (J) or unpaired *t*-test in (G) and (I). Statistical significance denoted as not significant (ns), *P < 0.05, **P < 0.01, ***P < 0.001, ****P < 0.0001.

LIPSTIC directly labels APCs that interact with naïve CD4^+^ T cells in an antigen-dependent manner, avoiding confounding factors owing to parallel immune responses to other luminal stimuli. In the LIPSTIC system, the CD40L, expressed by recently activated T cells (22), and its receptor CD40 are functionalized by fusing the proteins to Sortase A (SrtA) and its receptor G5, respectively. Injection of labeled SrtA substrate (the peptide LPETG conjugated to biotin) leads to its capture by donor SrtA-expressing T cells. APC-T cell interaction results in covalent transfer of substrate from T cell to APC. Interacting APCs are marked by biotin (biotin^+^) and, thus, detectable by flow cytometry with biotin specific reagents (Fig. 1A). APCs not presenting antigen recognized by SrtA-expressing T cells will be detected as biotin^*−*^.

To identify APCs that present dietary antigens and prime cognate naïve CD4^+^ T cells in adult mice, we adoptively transferred 10^6^ naïve ovalbumin (OVA)-specific CD40L-SrtA OT-II (*Cd40lg*^SrtA/Y^ OT-II) T cells, which recognize an MHC-II-restricted OVA peptide, into G5-CD40-expressing (*Cd40*^G5/G5^) recipient mice. These mice were given a single intragastric (i.g.) dose of OVA 18 hours after cell transfer, a protocol that induces OVA-specific pTregs capable of preventing the induction of future anaphylactic responses to that antigen (3). Since these OVA-specific pTregs are mainly generated in the duodenal gLNs (D-gLNs) (3), we focused our analysis on these LNs. LIPSTIC labeling of interacting APCs in the gLNs was carried out by intraperitoneal (i.p.) injections of biotinylated LPETG substrate 22–24 h after i.g. OVA (Fig. 1B), a point at which CD40L was upregulated in transferred OT-II cells (fig. S1, A and B). LIPSTIC labeled a small fraction (approximately 2–3%) of all DCs in the gLNs of mice that received OVA but not in PBS controls (Fig. 1C and fig. S1, C to F). Increasing the number of transferred OT-II T cells above that of 10^6^ did not increase DC labeling frequency, indicating that most or all DCs capable of presenting OVA to OT-II T cells were captured by this method (fig. S1G). Furthermore, DC labeling was fully inhibited by injection of a CD40L-blocking antibody (clone MR1) 22 hours prior to substrate injection, confirming that the CD40-CD40L interaction was required for labeling to take place (Fig. 1C). To evaluate whether DC-T cell interactions identified by LIPSTIC were cognate and dependent on DC MHC-II antigen presentation, we reconstituted lethally irradiated C57BL/6 mice with a mixture of *Cd40*^G5/G5^*.H2*^*+/+*^ and *Cd40*^G5/G5^*.H2*^*−/−*^ (MHC-II-deficient) bone marrow (Fig. 1D). Labeling was observed only in DCs that expressed MHC-II, confirming that the interactions captured by LIPSTIC at this time point were dependent on cognate antigen presentation (Fig. 1E). Thus, LIPSTIC provided an efficient method to identify the majority of DCs capable of presenting OVA antigen upon oral administration.

To assess the functional capacity of the DCs identified by LIPSTIC, we co-cultured 150 *Cd40*^G5/G5^ gLN biotin^+^ DCs with 750 naïve CD4^+^ OT-II CFSE-labeled T cells. Without the addition of exogenous OT-II peptide to the cultures, cell-sorted biotin^+^ DCs, but not biotin^−^ controls, were able to induce T cell proliferation and expression of Foxp3 (Fig. 1, F and G). Whereas both biotin^+^ and biotin^−^ DCs induced T cell proliferation in the presence of exogenous OT-II peptide, only biotin^+^ DCs were capable of inducing Foxp3^+^ Treg differentiation (Fig. 1H). Biotin^−^ and biotin^+^ DCs displayed the same survival rate at different timepoints in culture (fig. S1H). Therefore, DCs capable of presenting dietary OVA *in vivo* had intrinsic ability to induce a regulatory T cell response, at least under culture conditions. Corroborating previous studies (3,6, 12), LIPSTIC labeling was detected in DCs with a migratory (MHC-II^hi^) but not a resident (MHC-II^int^) phenotype (Fig. 1I). Despite the higher frequency of total cDC1s compared to cDC2s in the proximal duodenal (D)-gLNs, the preferential site for food-specific pTreg induction (3), labeling of cDC1 (CD103^+^CD11b^−^) and cDC2 (CD103^+/−^ CD11b^+^) by OT-II T cells occurred at a similar ratio (Fig. 1J and fig. S1, D to F). Therefore, both major migratory subsets of DCs were equally capable of presenting dietary antigens to specific CD4^+^ T cells. OVA-presenting DCs showed a similar profile when OVA was provided *ad libitum* in solid food rather than via oral gavage (fig. S1, I and J). We concluded that LIPSTIC was a sensitive and specific tool for monitoring intestinal cell-cell receptor-ligand interactions *in vivo* and accurately identified DCs that present antigen to dietary antigen-specific CD4^+^ T cells in gLNs.

### Biotin^+^ cDC1 contribute to food-specific pTreg differentiation

**Figure 2.**
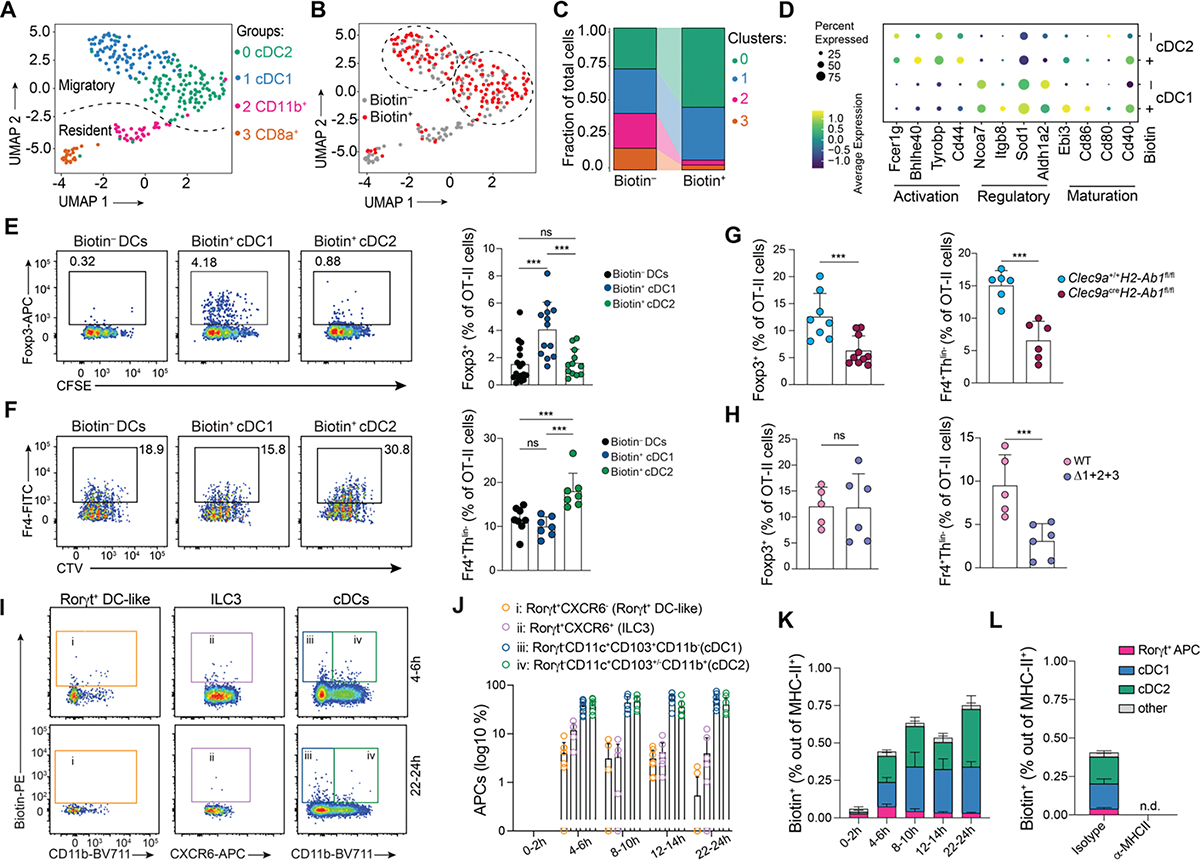
Biotin+ cDC1 contribute to food-specific pTreg differentiation. (A to F) CD45.2 *Cd40*^G5/G5^ mice were adoptively transferred with 1 × 10^6^ naive CD45.1 CD4^+^ *Cd40lg*^SrtA/Y^ OT-II T cells prior to one dose of intragastric OVA. Cell-cell interaction was revealed by LIPSTIC protocol 24 h later. Biotin^−^ and biotin^+^ D-gLNs DCs were single-cell sorted and subjected to scRNA-seq (263 cells were analyzed). **(A)** Uniform manifold approximation and projection (UMAP) plot. Cells were pooled from 7 mice from 2 independent experiments. Dotted line indicates the location of resident/migratory DC boundary. **(B)** Distribution of biotin^−^ (grey) and biotin^+^ (red) DCs. Dotted lines indicate the location of Cluster 0 and 1. **(C)** Proportion of cells in each transcriptional cluster among biotin^−^ and biotin^+^ DCs. **(D)** Expression of genes significantly upregulated in both biotin^+^ cDC1 and cDC2 compared to biotin^−^ cDC1 and cDC2. **(E, F)** OT-II CFSE- or CTV-labeled T cells were co-cultured *in vitro* with sorted biotin^−^ DCs, biotin^+^ cDC1s or biotin^+^ cDC2s from D-gLNs in the presence of exogenous OT-II peptide for 96 h. Representative flow plots showing percentage of Foxp3^+^ or Fr4^+^Th^lin−^ cells among proliferated OT-II T cells (left), and quantification of data (right). Each dot represents one mouse (n = 3, 4 mice per group, pool of four independent experiments). **(G, H)** CD45.2 *Clec9a*^+/+^*H2-Ab1*^*fl/fl*^ or *Clec9a*^cre^*H2-Ab1*^*fl/fl*^ mice; or bone marrow chimera (BMC) mice reconstituted with C57BL/6 (WT) or Δ1+2+3 cells were adoptively transferred with 1 × 10^6^ naive CD45.1 CD4^+^ OT-II T cells. Mice received two doses of intragastric OVA 48 h and 24 h prior analysis. Percentage of Foxp3^+^ or Fr4^+^Th^lin−^ cells among CD45.1 TCRV 2^+^ (OT-II) T cells in D-gLNs (n = 3 mice per group, pool of two or three independent experiments). **(I to L)** CD45.2 *Rosa26*^uLIPSTIC/uLIPSTIC^ mice were adoptively transferred with 3 × 10^6^ naive CD45.1 *Cd4*^Cre^*.Rosa26*^uLIPSTIC/uLIPSTIC^ OT-II T cells prior to one dose of intragastric OVA. Cell-cell interaction was revealed by LIPSTIC protocol at indicated time-points post oral OVA administration. **(I)** Representative flow plots showing percentage of labeled APCs in the D-gLNs at 4–6 h or 22–24 h after i.g. OVA. **(J, K)** Percentage of different APCs Biotin^+^ cells among MHC-II^+^ cells. **(L)** Percentage of Biotin^+^ APCs 4–6h post i.g. OVA with or without MHC-II blocking. (n = 3, 4 mice per group, pool of two or three independent experiments). cDC1s were defined as Aqua^−^ CD45.2^+^ CD45.1^−^ Lin^−^CD11c^hi^ MHC-II^hi^ CD103^+^ CD11b^−^ and cDC2s were defined as Aqua^−^ CD45.2^+^ CD45.1^−^ Lin^−^ CD11c^hi^ MHC-II^hi^ CD103^+/−^ CD11b^−^. In all graphs, the height of bars indicate mean, and error bars indicate SD. Wilcoxon signed-rank test was used for (D). *P*-values were calculated by one-way ANOVA in (E) and (F), or by unpaired *t*-test in (G) and (H). Statistical significance denoted as not significant (ns), ***P < 0.001. N.d = not detected.

To further characterize food antigen-presenting DCs, we performed plate-based single-cell mRNA sequencing (scRNA-seq) of biotin^+^ and biotin^−^ DCs from the duodenal gLNs (D-gLNs) 24 hours after i.g. OVA administration (fig. S2A). DCs fell into four transcriptional clusters (Fig. 2A), two corresponding to migratory DCs (Clusters 0 and 1) and two to resident DCs (Clusters 2 and 3), as determined by expression of *Ccr7* and MHC-II, both known to be highly expressed by migratory DCs (fig. S2B and C, and Data file S2). Among migratory DCs, Cluster 0 expressed signatures consistent with a cDC2 phenotype, whereas Cluster 1 DCs displayed a cDC1 phenotype (fig. S2D to F). In agreement with flow cytometry data, biotin^+^ DCs were composed of an approximately 1:1 mixture of cDC1s to cDC2s (Fig. 2B and C). cDC1s showed increased expression of several genes associated with pTreg generation, including *Aldh1a2* (RALDH2), *Tgfb2* (cytokine TGF-β2), *Itgb8* (integrin β8), *Ncoa7*, and *Sod1*(23, 24) (Fig. 2D and fig. S2F). In contrast, cDC2s were characterized by higher expression of genes associated with T cell activation *(Cd44, Tyrobp, Bhlhe40, Fcer1g*) (25–28) (Fig. 2D). Biotin^+^ cDC1s and cDC2s upregulated markers of DC maturation (*Cd80*, *Cd86*, *Cd40*) and the cytokine subunit *Ebi3* when compared to biotin^−^ cells (Fig. 2D and fig. S2G, Table S1), which we confirmed by flow cytometry (fig. S2, H to J). This upregulation was also observed when OVA was provided in solid food (fig. S2, K to M). To evaluate the ability of each biotin^+^ DC subset to induce pTregs, we co-cultured biotin^+^ cDC1s or biotin^+^ cDC2s with naïve CD4^+^ OT-II T cells, with or without addition of exogenous OT-II peptide. Only biotin^+^ cDC1s were capable of inducing pTreg differentiation above background levels (Fig. 2E and fig. S3A). Notably, biotin^+^ cDC2s more efficiently induced a recently-described noncanonical hyporesponsive T helper cell subset (called Fr4^+^Th^lin−^ cells), which has been proposed to play a role in tolerance to food due to their lack of inflammatory function and capacity to further differentiate into Tregs (29) (Fig. 2F and fig. S3B). cDC1s were also able to induce Fr4^+^Th^lin−^ cells, although less efficiently. OT-II T cells did not differentiate into Th1 (Tbet^+^), Tfh (CXCR5^+^), Th17 (Rorγt^+^ Foxp3^−^ or double-positive Foxp3^+^Rorγt^+^ Treg cell fates *in vivo* or when co-cultured with biotin^+^ cDC1s or cDC2s *in vitro* (fig. S3, B to F).

To confirm that cDCs, especially those of the cDC1 lineage, contributed to pTreg induction *in vivo* in adult animals, we targeted cDC subsets using specific mouse intersectional genetics approaches. To abrogate antigen presentation by cDC1s, we used mice carrying MHC-II floxed alleles crossed to mice expressing Cre recombinase under the *Clec9a* promoter (*Clec9a*^Cre^*H2-Ab1*^fl/fl^), which is active in all cDC1s, and in a fraction of cDC2s (15) (fig. S3, G to J). *Clec9a*^Cre^*H2-Ab1*^fl/fl^ mice received an adoptive transfer of naïve OT-II T cells, followed by two doses of i.g. OVA. OT-II pTreg induction was reduced by roughly 50% in the absence of antigen presentation by cDC1s (Fig. 2G). To specifically target cDC2s, we used *Zeb2* enhancer triple mutant (Δ1+2+3) mice, in which ablation of *Zeb2* expression impairs cDC2, but not cDC1 development (28). In these experiments, Δ1+2+3 mice were used as donors for total bone marrow (BM) transplant into lethally irradiated C57BL/6 mice hosts, generating chimeras rendering their hematopoietic compartment deficient in cDC2. In contrast to the reduced Treg induction observed in OT-II cells transferred to *Clec9a*^Cre^*H2-Ab1*^fl/fl^ mice, C57BL/6 mice reconstituted with WT or Δ1+2+3 bone marrow showed comparable frequencies of OT-II pTreg induction (Fig. 2H). Fr4^+^Th^lin−^ induction was reduced in both mouse models, suggesting that both cDC1s and cDC2s diverted T cells towards this phenotype (Fig. 2G and H). Our data suggested that, although both cDC subsets actively prime food-specific CD4^+^ T cells, only cDC1s play a role in pTreg differentiation towards dietary antigens.

The partial effect of MHC-II ablation and lack of antigen presentation by cDC1s on pTreg generation suggested that other, non-classical DC or APCs may also contribute to this process. Accordingly, recent studies have shown that Rorγt^+^ APCs are required for microbiota- (13–15) as well as diet-specific pTreg induction, especially in early life (30–32). To understand whether dietary-antigen presentation by this population cooperated with presentation by cDC1s, we sought to obtain an unbiased view of the cellular interactome of CD4^+^ T cells as they responded to a tolerogenic stimulus using universal (u)LIPSTIC system, which allows labeling of physically interacting cells regardless of the receptor-ligand pairs involved in the interaction (21). In this system, the SrtA is expressed in a Cre-dependent manner (i.e., CD4-cre will turn on SrtA expression in all T cells into uLIPSTIC donors) whereas all cre-negative cells express the uLIPSTIC acceptor G5 on their membrane, as SrtA or G5 are inserted in the ubiquitously expressed *Rosa26* locus.

We adoptively transferred 3×10^6^ naïve Cd4-cre.*Rosa26*^uLIPSTIC/uLIPSTIC^ OT-II CD4^+^ T cells into G5-expressing acceptors (*Rosa26*^uLIPSTIC/uLIPSTIC^) mice. These mice were given a single i.g. dose of OVA 18 h after cell transfer, and LIPSTIC labeling of interacting APCs in the gLNs was carried out by i.p. injections of biotinylated LPETG substrate 22–24 h after i.g. OVA (fig. S4A). In 14-day-old mice, in which Rorγt^+^ APCs are more frequent (13), around 0.75% of APCs were labeled with biotin in duodenal LNs, and several APC subsets were found to interact with OT-II T cells, including Rorγt^+^ APCs and cDCs (fig. S4, B to E). In adult mice, on average 0.60% of APCs were labeled with biotin, and cDCs were found to be the major interacting partner of OT-II T cells, representing over 95% of APCs presenting dietary OVA (fig. S4, B to E). These results also indicated that migratory cDCs were by far the major APC population presenting dietary antigen in adult mice 24 h post OVA gavage. To further investigate this dearth of labeling by Rorγt^+^ APCs in adult mice, we repeated the same experiment with labeling at different time-points after oral antigen delivery. LIPSTIC labeling of APCs was detected as early as 4 h post-gavage, when approximately 0.5% of all APCs were labeled in an MHC-II-dependent manner (Fig. 2, I to L). Rorγt^+^ APCs represented 20% of all interacting cells at 4 h post-gavage, and this frequency dropped progressively with time (Fig. 2, I to L). Conversely, the frequency of labeled cDCs increased with time, reaching 95% of OVA-presenting APCs at the 24 h timepoint. These data suggested a sequence of events in dietary-antigen presentation in which Rorγt^+^ APCs played an earlier role, possibly providing an early signal that primes naïve T cells towards a regulatory phenotype, followed by later engagement of cDCs in a CD40-CD40L dependent manner.

### Helminthic infections affect food-specific pTreg induction by DCs

**Figure 3.**
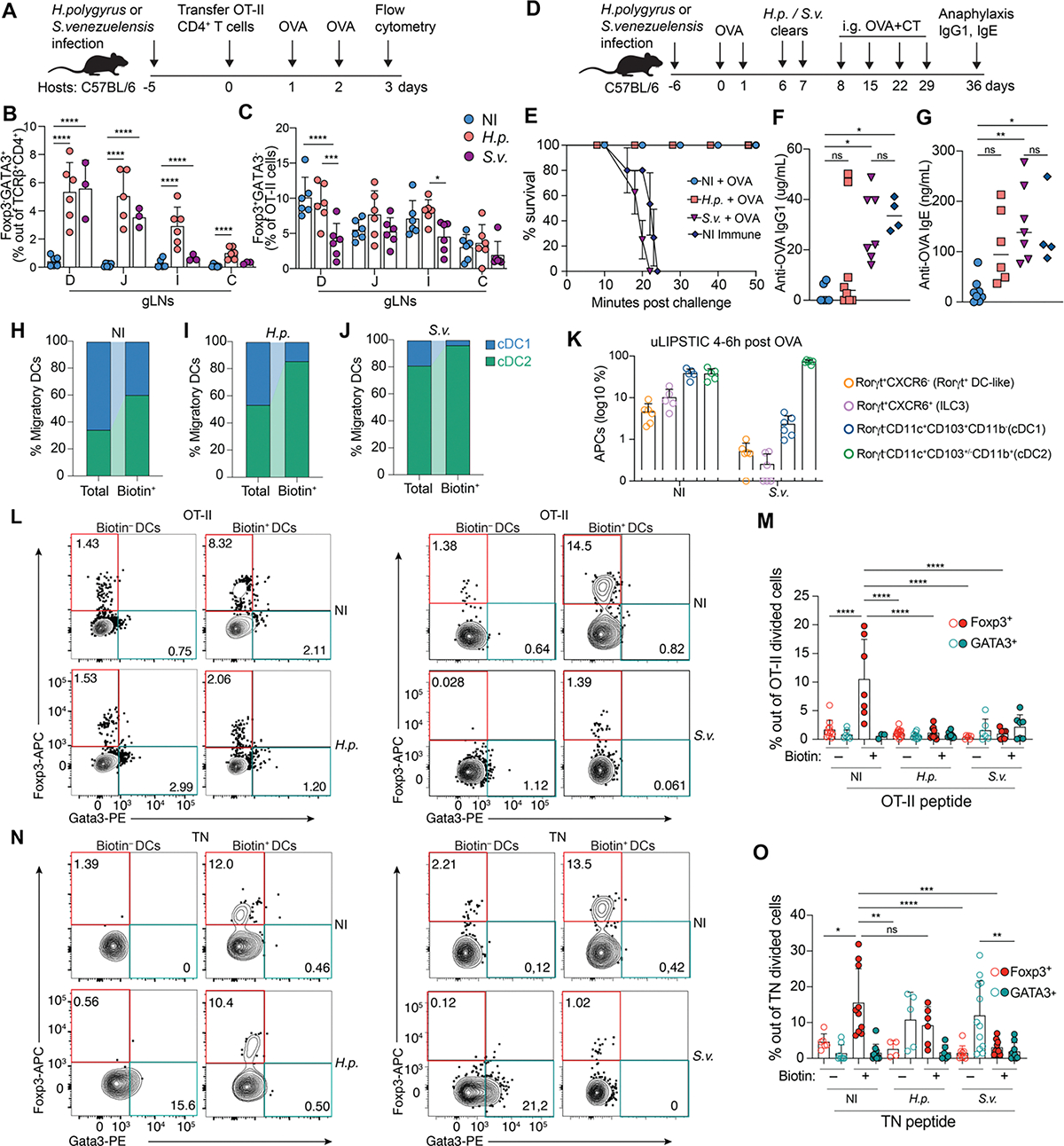
Helminthic infections affect food-specific pTreg induction by DCs. (A to C) CD45.2 C57BL/6 mice were infected with *S. venezuelensis (S.v.)* or *H. polygyrus (H.p.)* 5 days prior adoptively transfer of 1 × 10^6^ naive CD45.1 CD4^+^ OT-II T cells. Mice received two doses of intragastric OVA 48 h and 24 h prior analysis. Non-infected mice (NI) were used as control. **(A)** Experimental setup for panel (B, C). Percentage of **(B)** GATA3^+^ cells among CD45.2 TCRβ^+^CD4^+^ T cells and **(C)** Foxp3^+^ cells among CD45.1 TCRVα2^+^ (OT-II) T cells in gLNs. (n = 3 mice per group, pool of two independent experiments). **(D)** Experimental setup for panel (E-G). **(E)** Anaphylaxis as measured by survival of mice at the indicated times after intraperitoneal OVA injection (challenge), following four weekly doses of OVA+cholera toxin (CT). **(F)** OVA-specific IgG1 or **(G)** OVA-specific IgE levels in serum as measured by ELISA (n = 3, 4 mice per group, pool of three independent experiments). **(H to J)** CD45.2 *Cd40*^G5/G5^ mice were adoptively transferred with 1 × 10^6^ naive CD45.1 CD4^+^ *Cd40lg*^SrtA/Y^ OT-II T cells prior to one dose of intragastric OVA. Cell-cell interaction was revealed by LIPSTIC protocol 24 h later. Non-infected mice (NI) were used as control. Percentage of cDC1 and cDC2 out of total migratory DCs (left bar) and percentage of cDC1 and cDC2 out of biotin^+^ DCs (right bar) of NI, *H.p*- or *S.v*-infected mice. **(K)** CD45.2 *Rosa26*^uLIPSTIC/uLIPSTIC^ mice were infected with *S.v.* 5 days prior adoptively transfer of 3 × 10^6^ naive CD45.1 *Cd4*^Cre^*.Rosa26*^uLIPSTIC/uLIPSTIC^ OT-II T cells. Mice received one dose of intragastric OVA and cell-cell interaction was revealed by LIPSTIC protocol 4 h later. **(K)** Percentage of Biotin^+^ APCs among MHC-II^+^ cells. **(L to O)** Sorted biotin^−^ or biotin^+^ DCs from D-gLNs of non-infected mice (NI) or mice infected with *H.p* (left) or *S.v* (right) were co-culture with OT-II CFSE-labeled T cells **(L, M)** or TN CFSE-labeled T cells **(N, O)** *in vitro* for 96 h. **(L, N)** Representative flow plots showing percentage of Foxp3^+^ and GATA3^+^ cells among proliferated T cells co-cultured with biotin^−^ or biotin^+^ DCs from NI, *H.p* (left) or *S.v* (right) infected mice and **(M, O)** quantification of data. OT-II or TN peptide were added to the corresponding co-cultures wells. Each dot represents one mouse (n = 3, 4 mice per group, pool of three independent experiments). cDC1s were defined as Aqua^−^ CD45.2^+^ CD45.1^−^ Lin^−^ CD11c^hi^ MHC-II^hi^ CD103^+^ CD11b^−^ and cDC2s were defined as Aqua^−^ CD45.2^+^ CD45.1^−^ Lin^−^ CD11c^hi^ MHC-II^hi^ CD103^+/−^ CD11b^−^. Height of bars indicate mean, and error bars indicate SD. *P*-values were calculated by two-way ANOVA in (B), (C), (F), (G), (M) and (O). Statistical significance denoted as not significant (ns), *P < 0.05, **P < 0.01, ***P < 0.001, ****P < 0.0001.

We previously showed that infection with *Strongyloides venezuelensis (S.v.)*, a helminth with duodenal tropism, prevents localized food-specific pTreg development and impairs the induction of oral tolerance by generating an immunological conflict at the site of oral antigen presentation in D-gLNs (3). To determine whether other helminth infections could interfere with food-specific pTreg differentiation and oral tolerance, we infected C57BL/6 mice with *Heligmosomoides polygyrus (H.p.)*, which also displays duodenal tropism but is of more symbiotic nature in mice than *S.v.*, establishing chronic infection and inflammation (33). Eight days post-infection (d.p.i), the peak of response to *S.v.*, which is cleared around 14 d.p.i., the frequencies of inflammatory Th2 cells (expressing GATA3^+^) in the gLNs of *H.p.*- and *S.v.*-infected mice were similar (Fig. 3, A and B). We adoptively transferred naïve OT-II T cells into host mice at 5 d.p.i, followed by two doses of i.g. OVA (Fig. 3A). OT-II pTreg induction was drastically reduced in mice infected with *S.v.* but was only slightly diminished by *H.p*. infection (Fig. 3, A and C). Whereas an orally administered antigen alone leads to oral tolerance, co-administration of the same antigen with cholera toxin (CT) can elicit allergic sensitization. In this food allergy model, administration of OVA+CT results in anaphylaxis, with a sudden drop in body temperature and eventually, death (34). Accordingly, while oral OVA protected uninfected or *H.p.*-infected mice against OVA-induced food allergy (35) and asthma responses (16), *S.v.*-infected mice remained fully susceptible (Fig. 3, D to G and fig. S5, A to D). These effects of *S.v.* infection on the induction of pTregs and tolerance decreased after helminth clearance and were no longer noticeable at 28 days post-infection (fig. S5, E to I). Thus, prevention of OVA-specific pTreg generation occurred only during concomitant *S.v.* infection.

Finally, to assess whether *S.v.* infection affected Treg induction broadly, including diet- and helminth-specific Tregs, we used the i*Sell*^tomato^ fate-mapping approach, in which naïve T cells, which express high levels of CD62L (encoded by *Sell*), will be permanently labeled with the tomato protein upon tamoxifen administration. Ex-naïve activated T cells downregulate CD62L and therefore become “fate-mapped” Tomato^+^CD62L^−^ T cells, which are highly enriched in cells specific for the most recent antigenic stimulus (36). Indeed, 40% of Tomato^+^CD62L^−^ T cells after *S.v.* infection expressed GATA3^+^ (indicative of a Th2 phenotype), whereas this was true for only 3% of Tomato^−^ cells (fig. S5J). In contrast, Tomato^+^CD62L^−^ cells from *S.v.* infected mice expressed less Foxp3 in the lamina propria than fate-mapped cells from naïve mice (fig. S5K), suggesting that certain helminth infections impair Treg generation broadly at the peak of infection.

To investigate the cellular mechanisms by which *S.v.* but not *H.p.* infection abrogated oral tolerance, we infected *Cd40*^G5/G5^ mice with each helminth, adoptively transferred naïve *Cd40lg*^SrtA/Y^ OT-II T cells, followed by one dose of i.g. OVA and then performed LIPSTIC labeling 24 h later. Whereas roughly 15% of DCs presenting dietary antigens to CD4^+^ T cells during *H.p.* infection were cDC1s, this fraction fell to virtually zero in the D-gLNs of *S.v.*-infected mice (Fig. 3, H to J and fig. S6, A to D). DC-T cell co-culture experiments showed that biotin^+^ DCs from infected mice failed to induce pTregs *in vitro* in the presence of exogenous OT-II peptide (Fig. 3, L and M). Notably, *S.v.* infection also almost completely abrogated dietary-antigen presentation by Rorγt^+^ APCs and cDC1s at 4–6h post OVA gavage (Fig. 3K).

To assess the intrinsic ability of dietary antigen-presenting DCs to induce pTregs, we co-cultured OVA-presenting biotin^+^ DCs from D-gLN of *Cd40*^G5/G5^ mice, labeled by *Cd40lg*^SrtA/Y^ OT-II T cells *in vivo*, as well as biotin^−^ DCs, with monoclonal transnuclear (TN) T cells bearing an unrelated TCR that specifically recognized a peptide derived from commensals of the *Bacteroidetes* phylum (37), but not the dietary OVA antigen. We co-cultured biotin^+^ DCs with naïve CD4^+^ TN T cells in the presence of the TN cognate peptide. TN cells differentiated into pTregs when cultured with biotin^+^ DCs from uninfected or *H.p.*-infected mice, but not from *S.v.*-infected mice (Fig. 3, N and O). Thus, biotin^+^ DCs (i.e., those capable of presenting OVA *in vivo*) were able to induce pTreg differentiation *in vitro* when presenting an unrelated antigen, confirming that this was an intrinsic trait of the biotin^+^ DCs rather than a property of the specific antigen they acquired *in vivo*. In contrast, biotin^−^ DCs from both helminth infections induced TN cells to differentiate towards a GATA3^+^ Th2 phenotype (Fig. 3, N and O), in accordance with the *in vivo* findings of endogenous Th2 response to helminth infections (3, 28) (Fig. 3B). Of note, GATA3 expression was observed in TN but not OT-II T cells cultured under similar conditions, which may reflect different properties and activation thresholds of monoclonal T cells bearing different TCRs. We concluded that, although *H.p.* and *S.v.* infections have distinct effects on the ability of DCs to induce pTreg differentiation, DCs presenting dietary antigens did not promote an overt diet-specific Th2 response, even under conditions of strong type-2 inflammation.

### Helminth infection skews the populations of dietary antigen-presenting biotin^+^ DCs in the gLNs

**Figure 4.**
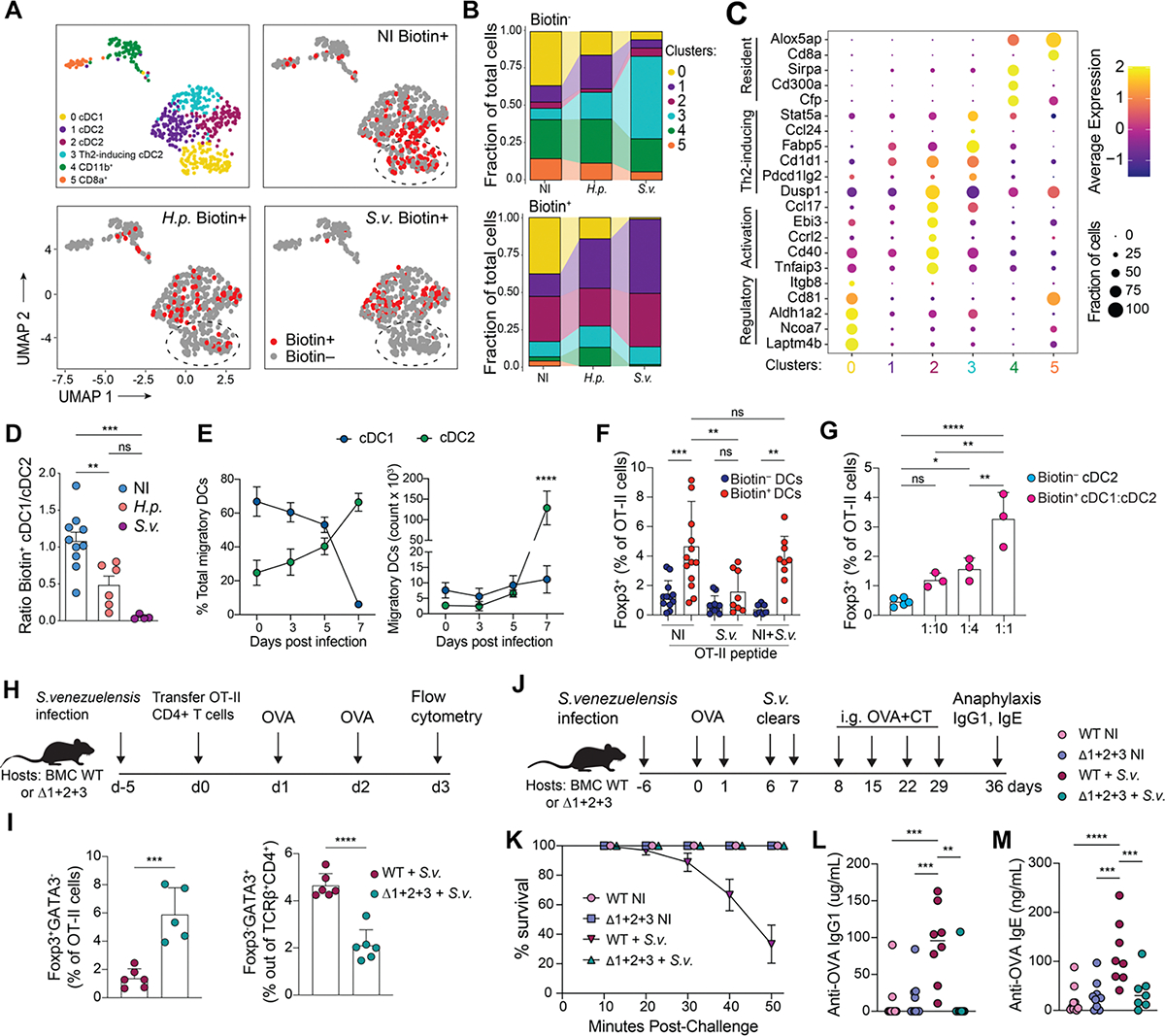
Helminth infection skews the population of dietary antigen-presenting biotin+ DCs in the D-gLNs. CD45.2 *Cd40*^G5/G5^ mice were infected with *S. venezuelensis (S.v.)* or *H. polygyrus (H.p.)* 5 days prior adoptive transfer of 1 × 10^6^ naive CD45.1 CD4^+^ *Cd40lg*^SrtA/Y^ OT-II T cells. Animals received 1 dose of intragastric OVA 18 h after OT-II T cells transfer. Cell-cell interaction was revealed by LIPSTIC protocol 24 h after OVA administration. Non-infected (NI) mice were used as control. **(A)** Biotin^−^_and biotin^+^ D-gLNs DCs were single-cell sorted and subjected to scRNA-seq (534 cells were analyzed). UMAP plot showing clustering of sequenced DCs of NI mice or mice infected with *H.p* or *S.v*. Cells were pooled from 4–7 mice from 2 independent experiments. Distribution of biotin^−^ (grey) and biotin^+^ (red) DCs in the same plot. Dotted lines indicate the location of Cluster 0. **(B)** Proportion of cells in each transcriptional cluster among biotin^−^ and biotin^+^ DCs. **(C)** Dot plot showing expression of genes differentially expressed between Clusters. **(D)** Ratio of cDC1/cDC2 among biotin^+^ DCs from D-gLNs of NI mice or mice infected with *H.p* or *S.v.* **(E)** Relative frequency (left) and absolute numbers (right) of migratory cDC1 and cDC2 in D-gLNs of naïve C57BL/6 mice or mice infected with *S.v.* **(F)** Percentage of Foxp3^+^ cells among proliferated OT-II CFSE-labeled T cells *in vitro* after 96 h of co-culture with D-gLN NI or *S.v* biotin^−^ or biotin^+^ DCs or a combination of NI and *S.v* DCs (1:1 ratio). **(G)** Percentage of Foxp3^+^ cells among proliferated OT-II CFSE-labeled T cells *in vitro* after 96 h of co-culture with D-gLN NI or *S.v*. biotin^+^ cDC1 and cDC2 at indicated ratios. OT-II peptide was added to the co-cultures. Each dot represents one mouse (n = 3 to 5 mice per group, representative of two independent experiments). **(H and I)** Bone marrow chimera (BMC) mice reconstituted with C57BL/6 (WT) or Δ1+2+3 cells. Mice were infected with *S.v.* 5 days prior adoptively transfer of 1 × 10^6^ naive CD45.1 CD4^+^ OT-II T cells. Mice received two doses of intragastric OVA 48 h and 24 h prior analysis. **(H)** Experimental set up for panel (I). **(I)** Percentage of Foxp3^+^ cells among CD45.1 TCRVα2^+^ (OT-II) T cells (right) and percentage of GATA3^+^ cells among CD45.2 TCRβ^+^CD4^+^ T cells (left) in D-gLNs (n = 3 mice per group, pool of two independent experiments). **(J)** Experimental setup for panel (K to M). **(K)** Anaphylaxis as measured by survival of mice at the indicated times after intraperitoneal OVA injection (challenge), following four weekly doses of OVA+cholera toxin (CT). **(L)** OVA-specific IgG1 or **(M)** OVA-specific IgE levels in serum as measured by ELISA (n = 3, 4 mice per group, pool of three independent experiments). cDC1s were defined as Aqua^−^ CD45.2^+^ CD45.1^−^ Lin^−^ CD11c^hi^ MHC-II^hi^ CD103^+^ CD11b^−^ and cDC2s were defined as Aqua^−^ CD45.2^+^ CD45.1^−^ Lin^−^ CD11c^hi^ MHC-II^hi^ CD103^+/−^ CD11b^−^. In graphs, the height of bars indicate mean, and error bars indicate SD. Wilcoxon signed-rank test was used for (C). *P*-values were calculated by one-way ANOVA in (D), or by two-way ANOVA in (F), (G), (L) and (M), and by unpaired *t*-test in (I). Statistical significance denoted as not significant (ns), *P < 0.05, **P < 0.01, ***P < 0.001, ****P < 0.0001.

To understand how different helminth infections impacted the phenotypes of dietary antigen-presenting DCs, we profiled single OVA-presenting DCs from the D-gLNs in the context of *H.p* and *S.v.* infection and compared these to our previous steady-state dataset (Fig. 2). Sequencing data pooled from these two experiments assigned DCs to 6 major clusters, four of which (0, 1, 2 and 3) corresponded to migratory DCs and two (4 and 5) to resident DCs (Fig. 4, A to C). Among migratory DCs, Cluster 0 cells expressed genes associated with a cDC1 signature, whereas Clusters 1, 2, and 3 were associated with a cDC2 phenotype (fig. S7, A and B, and Data file S3), in accordance with previous reports showing that cDC2s display a more heterogeneous transcriptional program (38, 39). Among biotin^−^ DCs, *S.v.* and, to a lesser extent, *H.p.* infection led to an expansion of cDC2s in Cluster 3, a cluster largely absent from the steady-state dataset (Fig. 4B).

Accordingly, DCs from this cluster were characterized by high expression of genes associated with a pro-inflammatory Th2 response to worm infections, such as *Pdcd1lg2* (which encodes PD-L2) (40–42), *Stat5a (43)*, *Ccl24* (a chemotactic factor for eosinophils and lymphocytes) (44), and *Cd1d1* (involved in lipid-based antigen presentation to iNKT cells, which play a role in the response to worms) (45) (Fig. 4C). Cluster 3 cDC2s expressed more pro-inflammatory genes than the Cluster 1 cDC2s that were expanded among biotin^+^ DCs in infected mice (Fig. 4C and fig. S7, C and D). Together with the capacity of biotin^−^ DCs to induce Th2 differentiation (see Fig. 3, N and O), these data suggested that Cluster 3 cDC2s were responsible for inducing a Th2 response against helminth infections, in agreement with previous studies (28). Notably, we found lower expression of MHC-II on total cDC2s of D-gLNs from *S.v.*-infected mice compared to uninfected mice (fig. S7E), suggesting a decreased capacity of *S.v*.-infected cDC2s to present soluble antigens. We did not detect differences in the frequency of Cluster 3 cells among biotin^+^ DCs in the three experimental groups, potentially explaining the absence of food-specific Th2 responses during helminth-induced type 2 immunity.

DCs from Cluster 0 displayed a tolerogenic transcriptional program (Fig. 4C) corresponding to that of the pTreg-inducing cDC1 population identified in Fig. 2. The ratio of cDC1 to cDC2 in the OVA-presenting biotin^+^ compartment, approximately 1 at steady state, decreased to 0.5 upon *H.p.* infection and approached zero in the presence of *S.v.* due to the low frequency of biotin^+^ cDC1s (Fig. 4D). By contrast, there was a gradual increase in Cluster 1 cDC2s among DCs presenting dietary antigens in the context of *H.p.* and, especially, *S.v.* infection, which corresponded with an increase in Fr4^+^Th^lin−^ OT-II cells (Fig. 4B and fig. S7F). This shift was due to an increase in cDC2 numbers between days 5 and 7 post-*S.v.* infection, whereas the number of other APCs were not affected (Fig. 4E and fig. S7G). To test whether cDC2 increase was caused by *in situ* proliferation, mice were treated from day 5 to 7 post-infection with EdU, which is incorporated into the DNA of actively dividing cells. EdU labeling showed lower incorporation by both cDC1s and cDC2s in *S.v.*-infected mice (fig. S7H), suggesting that the increase in D-gLN cDC2s was not due to preferential proliferation.

To determine whether helminth-induced changes in D-gLN DC populations were imprinted in the gut tissue, prior to DC migration to the gLNs, we profiled DCs from the duodenal lamina propria of uninfected, *H.p*.- or *S.v*.-infected mice and sorted them at 7 d.p.i., 1 day after i.g. OVA administration. Droplet-based scRNA-seq revealed a population of cDC2s present primarily in *S.v.*-infected mice and which displayed a pro-inflammatory Th2-inducing phenotype characterized by expression of genes also found in D-gLNs under these same conditions (fig. S8, A to D, and Data file S4). This confirmed that *S.v*. infection altered the profile of DCs already in the LP, suggesting that the increase in D-gLN cDC2s was due to an influx of these cells from the LP.

To assess whether an increase in the ratio of OVA-presenting cDC2s to cDC1s in the D-gLN could prevent Treg induction, we co-cultured naïve CD4^+^ OT-II T cells with DCs presenting OVA during *S.v.* infection (*S.v.* biotin^+^ DCs) and DCs presenting OVA at steady-state (OVA biotin^+^ DCs) either alone or in combination at a 1:1 ratio. Whereas *S.v.* biotin^+^ DCs failed to induce differentiation of pTregs, both OVA biotin^+^ DCs and the combination of *S.v.* and OVA DCs were able to do so (Fig. 4F). We then co-cultured naïve CD4^+^ OT-II T cells with a fixed number of total DCs, varying the ratios of OVA biotin^+^ cDC1s to *S.v.* biotin^+^ cDC2s. pTreg induction was optimal at a 1:1 cDC1:cDC2 ratio but decreased progressively so that no pTregs were induced at a cDC1:cDC2 ratio of 1:10 (Fig. 4G). Additionally, co-culture of naïve CD4^+^ OT-II T cells with *S.v.* biotin^+^ cDC1s (pooled from different mice due their very low numbers) induced similar frequencies of Treg differentiation as OVA biotin^+^ cDC1s (fig. S8E), suggesting that worm infection did not alter the tolerogenic nature of cDC1s. We concluded that pTreg induction by biotin^+^ cDC1s was highly sensitive to the ratio of tolerogenic to non-tolerogenic APCs.

Lastly, we sought to assess whether the loss of OVA-specific pTreg induction upon infection *in vivo* could also be attributed to a reduced cDC1 to cDC2 ratio. We infected WT and Δ1+2+3 bone marrow chimeras with *S.v.*, transferred naïve OT-II T cells at 5 d.p.i, and administered OVA i.g. one and two days later (Fig. 4H). Cells with a cDC2-phenotype were induced to some degree in the D-gLNs of *S.v.*-infected Δ1+2+3 chimeras, as shown previously with *H.p.* infection (28), such that the cDC1 to cDC2 ratio in this setting approached the steady-state ratio of 1 (fig. S9, A to C). Accordingly, OT-II pTreg induction was restored in Δ1+2+3 bone marrow hosts, whereas the endogenous Th2 response was reduced (Fig. 4I). Correspondingly, oral OVA completely protected *S.v*.-infected Δ1+2+3 bone marrow chimeras from anaphylaxis in a model of OVA-induced food allergy (Fig. 4, J to M). Altogether, these data suggested that, during tolerance-disrupting infections, tolerogenic antigen-presenting cDCs, and likely also Rorγt^+^ APCs, rather than losing their tolerogenic capacity, became largely excluded from engaging in antigen presentation to dietary antigen-specific T cells due to an increase in presentation by cDC2s.

## Discussion

The ability to isolate the exact APCs within a population that present antigens to the T cells of interest *in vivo* has been a longstanding challenge to the field. Adapting LIPSTIC for use in the intestine allowed us to identify individual APCs actively engaged in presenting dietary antigens and priming food-specific CD4^+^ T cells in gLNs. Hence, we were able to determine the abundance and kinetics as well as define the transcriptional and functional programs of these APCs at steady state and in the context of infection-mediated disruption of pTregs and oral tolerance induction.

Although our study focused on adult animals, observations made in young mice add support of a role for additional APC subsets, particularly Rorγt^+^ CXCR6^+^ (ILC3s) (15) Rorγt^+^ CXCR6^−^ (TCs or JCs) (13, 14, 46), in the induction of luminal-specific pTregs during the neonatal period. We detected low level antigen presentation by Rorγt^+^ APCs also in adults, which appears restricted to the initial hours following antigen delivery. A speculative model is that Rorγt^+^ APCs may provide early signals to T cells, possibly by capturing soluble antigen arriving at gLNs via lymphatics, prior to the arrival of migratory DCs that carry antigen acquired in the LP and provide further proliferative and/or differentiation signals.

*S.v.* infection prevented oral tolerance induction at least in part by abrogating dietary antigen presentation by cDC1s and Rorγt^+^ APCs, thus blocking pTreg generation. Our genetic cDC2 targeting during *S.v.* infection, aided by our LIPSTIC co-culture experiments, suggested that it is the ratio of cDC2s to cDC1s (and other tolerogenic APCs)—rather than an inhibitory effect of cDC2—that accounted for reduced pTreg differentiation during helminth infection. Whereas Th2-promoting cDC2s increased in abundance during infection, they are unable to present dietary antigens to an extent that would promote food-specific Th2 responses upon OVA feeding. It remains to be determined whether additional “immunological conflicts” such as reovirus infection, shown to induce food-specific Th1 responses (47, 48), or scarring downstream of bacterial infection, shown to prevent pTreg and oral tolerance induction (49), specifically interfere with dietary antigen presentation by cDC subsets.

LIPSTIC allowed us to uncover a compartmentalized antigen presentation setting that prevented the induction of food-specific Th2 cells, even in the presence of robust induction of type-2 immunity by helminth infections, which were previously shown to break T cell tolerance (50). Furthermore, the intestine-adapted LIPSTIC and uLIPSTIC technologies presented here might be useful for future investigations of *in vivo* APCs inducing regulatory and inflammatory T cell responses to commensals or enteric pathogen- derived antigens.

In conclusion, this study provides insight into the role of cDC1s in the induction of food-specific pTregs and oral tolerance, and uncovers a mechanism by which infection can disrupt this pathway to impair oral tolerance by exclusion of cDC1 and Rorγt^+^ APCs from antigen presentation to CD4^+^ T cells in draining LNs, rather than by their reprogramming (47, 48). Our findings illustrate how dynamic APC-T cell interactions drive tolerance or inflammation towards dietary proteins in the complex gut environment.

## Materials and methods

### Mice.

CD45.2 (C57BL/6) mice (strain number 000664), H2−/− mice (strain number 003584), *Foxp3*^RFP^ mice (strain number 008374) and CD4-Cre mice (strain number 022071) were purchased from the Jackson Laboratories. *Rosa26*^uLIPSTIC^ mice (Jackson Laboratories strain 038221) were generated and maintained in our laboratory (21). CD45.1 OT-II TCR-transgenic were originally purchased from Taconic Farms, Rag1^−/−^ was bred out, and mice were maintained in our facilities (strain number 4234-M). *Cd40*^G5/G5^ mice (Jackson Laboratories strain 037113) were generated and maintained in our laboratory (20). *Clec9a*^cre^*H2-Ab1*^fl/fl^ mice were provided by Gregory Sonnenberg (Weill Cornell Medicine). D1+2+3 mice were provided by Kenneth Murphy (Washington University in St. Louis). Transnuclear (TN) mice were generated as described (51) and maintained in our facilities. *Sell*^Cre-ERT2^ mice were provided by M. Nussenzweig (52), crossed with Rosa ^26CAG-LSL-tdTomato-WPRE^ (007914) mice from Jackson Laboratory, and maintained in our facilities. Male and female young mice at 14 days old (P14) or adult mice at 8–12 weeks old were used throughout the study. Mice were maintained at the Rockefeller University animal facilities under specific pathogen-free conditions. Mice were euthanized by cervical dislocation. All protocols were approved by the Rockefeller University Institutional Animal Care and Use Committee.

### Generation of *Cd40lg*^SrtAv2^ mice.

*Cd40lg*^SrtAv2^ mice (Jackson Laboratories strain 037113) were generated in our laboratory using the Easi-CRISPR method(53). Cas9-crRNA-tracrRNA complexes targeting the last exon of the *Cd40lg* locus (protospacer sequence, GAGTTGGCTTCTCATCTTT) were microinjected along with a single-stranded DNA templates encoding a C-terminal SrtA fusion flanked by 200 bp homology arms into the pronuclei of fertilized C57BL6 embryos, which were then implanted into pseudopregnant foster dams. Founder mice were backcrossed to WT C57BL6 mice for at least 5 generations to reduce the probability of transmitting CRISPR-induced off-target mutations.

### Reagents.

Ovalbumin (grade III, A5378; grade VI, A2512) and Pyrantel Pamoate (P6210) were purchased from Sigma. LPS-free ovalbumin was from Hyglos, Germany (Cat. no 77161). Cholera toxin was purchased from List Biological (Cat. No 100B). 5-Ethynyl-20-deoxyuridine (EdU) and Click-iT Plus EdU Alexa Fluor 647 Flow Cytometry Assay Kit were purchased from Thermo Fisher Scietific (Cat. No A10044 and C10634).

### Flow cytometry.

Cells were stained with reagents listed in Data S1. Cell populations were stained with Aqua or Zombie in PBS, followed by incubation with Fc block and antibodies against the indicated cell surface markers in FACS buffer (PBS, 1% BSA, 10 mM EDTA, 0.02% sodium azide). The cells were analyzed live or fixed in 1% PFA/PBS. For intracellular staining, cells were first stained for surface epitopes and then fixed, permeabilized and stained according to the manufacturer’s protocol (eBioscience 00–5123-43). Unless otherwise stated, we used the following gating: cDC1s Aqua^−^ CD45.2^+^ CD45.1^−^ Lin^−^ (TCRb^−^B220^−^CD64^−^) CD11c^hi^ MHC-II^hi^ CD103^+^ CD11b^−^; cDC2s: Aqua^−^ CD45.2^+^ CD45.1^−^ Lin^−^ CD11c^hi^ MHC-II^hi^ CD103^+/−^ CD11b^−^; Rorγt DC-like: Aqua^−^ Lin^−^ (SIGLEC-F, TCRβ, TCRγδ, CD19, B220, NK1.1, CD64^−^) Ly6C^−^ MHCII^+^ RORγt^+^ CXCR6^−^; MHCII^+^ ILC3s: Aqua^−^ Lin^−^, CD64^−^Ly6C^−^ RORγt^−+^ CXCR6^+^ MHCII^+^. Flow cytometry was performed on FACSymphony A5 (BD Biosciences) and analyzed using FlowJo Software package (Tri-Star).

### SrtA substrate.

Biotin-aminohexanoic acid–LPETGS (C-terminal amide, 95% purity) was purchased from LifeTein (custom synthesis) and stock solutions prepared in PBS at 20 mM.

### LIPSTIC *in vivo*-labeling experiments.

Biotin-LPETG substrate was injected into *Cd40*^G5/G5^ or *Rosa26*^uLIPSTIC^ mice intraperitoneally (i.p.) (100 μl of 20 mM solution in PBS) six times 20 min apart. gLNs were collected 40 min after the last injection. Mice were briefly anaesthetized with isoflurane at each injection. For CD40L-blockade experiments, mice were injected intravenously with 200 μg of CD40L-blocking antibody (clone MR-1, BioXCell) 22 h prior to substrate injection. For MHC-II-blockade experiments, mice were injected intraperitoneally with 400 μg of MHC-II-blocking antibody (clone M5/114, BioXCell) 14 h prior to OVA gavage and a second dose of 200 μg of MHC-II-blocking antibody at the time of OVA gavage.

### Lymphocyte and dendritic cell isolation from lymph nodes.

Tissues were dissected into cold RPMI supplemented with 10% heat-inactivated fetal bovine serum (Hyclone), 2 mM L-glutamine, 100 units per ml of penicillin, 100 μg/ml of streptomycin sulfate, 1 mM sodium pyruvate, 0.1 mM non-essential amino acids, 10 mM HEPES (all from Gibco), and 50 μM β mercaptoethanol (Sigma). Lymph nodes were finely chopped and incubated in 400 U/ml Collagenase D (Roche) in supplemented RPMI for 25 min at 37°C, 5% CO2. Single cell suspensions were extracted from connective tissue by taking up and resuspending the digests five times.

### Segmentation of gut-draining lymph nodes.

The mouse gLNs consist of one hepatic–coeliac lymph node co-draining the duodenum, pancreatic–duodenal lymph nodes draining the duodenum and separately the ascending and transverse colon, the main mLN chain draining the distal duodenum, jejunum, ileum, caecum and proximal ascending colon, and the caudal and iliac lymph nodes draining the descending-distal colon. Mesenteric lymph nodes draining intestinal segments were identified anatomically by following the lymphatic vessels connecting the colon, ileum and jejunum to their lymph nodes. Duodenal lymph nodes were revealed by gavaging with 100 μl of olive oil (Sigma) and determining the most stomach-proximal lymph nodes surrounded by chyle, indicative of duodenal drainage, 1 h after gavage, as described before (3).

### Dendritic cell isolation from duodenum lamina propria.

Intestines were separated from the mesentery, and Peyer’s patches and feces were removed. For segmentation of the small intestine, the upper 25% of the small intestine was taken as the duodenum, as described previously (3). Intestines were cut longitudinally and washed twice in PBS. Tissue was cut into 1-cm pieces, mucus was removed by incubating the tissue for 10 min in PBS and 1 μM DTT, and the epithelium was removed by two incubations in 25 ml of RPMI, 2% FCS and 30 mM EDTA for 10 min at 37 °C at 230 r.p.m. with vigorous shaking after each incubation. Tissues were washed in PBS over a sieve, then finely chopped and digested in 6 ml of RPMI, 2% FCS, 200 μg/ml DNaseI (Roche) and 2 mg/ml collagenase 8 (Gibco) for 45 min at 37 °C, 5% CO2. Digests were taken up and resuspended 10 times and passed through a sieve, and collagenase was quenched by addition of 15 ml cold RPMI, 2% FCS. Cell pellets were resuspended in 40% Percoll (BD Pharmigen) complemented with RPMI, 2% FCS, passed through a 100-μm mesh and separated by centrifugation in a discontinuous Percoll gradient (80%/40%) at 1000g for 25 min at room temperature. DCs were isolated from the interphase, washed, and stained for FACS bulk sorting.

### Single-cell sorting.

Dendritic cells were collected, as described above, from the duodenum-gLN of *Cd40*^G5/G5^ male mice (8–12 weeks old). Single cells from three to four mice per each condition were index-sorted directly into 96-well plates containing 5μl of TCL buffer (Qiagen) supplemented with 1% β-mercaptoethanol using a BD FACS Aria II instrument. After sorting, plates were immediately frozen on dry ice and stored at −80°C before processing. All sorting data were analyzed using FlowJo software v.10. Dendritic cells were sorted as Aqua^−^CD45.2^+^CD45.1^−^Lin^−^ (TCRb^−^B220^−^CD64^−^)CD11c^hi^MHC-II^int/hi^Biotin^−^ or Biotin^+^. Dendritic cells were also stained for, but not sorted based on, CD103, CD11b and CD8a for index analysis.

### Bulk sorting.

Dendritic cells were collected, as described above, from the duodenum-gLN of bone marrow chimera mice reconstituted with C57BL/6 (WT) or D1+2+3 cells. Three hundred cells from three mice per condition were sorted directly into 25 μl TCL buffer (Qiagen, 1031576) supplemented with 1% β-mercaptoethanol at single cell precision using a BD FACS Aria II instrument. After sorting, samples were immediately frozen on dry ice and stored at −80°C before processing. Dendritic cells were sorted as Aqua^−^CD45.2^+^Lin^−^(TCRb^−^B220^−^CD64^−^)CD11c^hi^MHC-IIint/hi.

### Library preparation for single-cell RNA-seq (Smartseq2).

Each sorted plate contained all conditions assayed in each replicate and libraries were prepared as previously described^41^. RNA was extracted from single cells using RNAClean XP Solid Phase Reversible Immobilization (SPRI) beads (Agentcourt, Beckman Coulter), and hybridized first using RT primer (5′/5Biosg/AAGCAGTGGTATCAACGCAGAGTACTTTTTTTTTTTTTTTTTTTTTTTTTTTTTTVN-3′) and then reverse transcribed into cDNA using TSO primer (5′-AAGCAGTGGTATCAACGCAGAGTACATrGrGrG-3′) and RT maxima reverse transcription (Thermo Fisher Scientific). cDNA was amplified using ISPCR primer (5′-AAGCAGTGGTATCAACGCAGAGT-3′) and KAPA HiFi HotStart ReadyMix (Thermo Fisher Scientific), cleaned up using RNAClean XP SPRI beads three times, and tagmented using Nextera XT DNA Library Preparation Kit (Illumina) following manufacturer’s instructions. For each sequencing batch, up to four plates were barcoded at a time with Nextera XT Index Kit v2 Sets A– D (Illumina). Finally, barcoded libraries were pooled and sequenced using Illumina Nextseq 550 platform.

### Library preparation for single-cell RNA-Seq (10x Genomics).

Cells were first co-stained with hashtag oligonucleotide (HTO) - labeled CD45 and MHC-I antibodies prior to the sorting process for sample separation, with two hashtags per sample. Sorted cells were collected in a microfuge tube containing 300 μl PBS 0.4% BSA. Following sorting, tubes were topped with PBS 0.4% BSA, centrifuged, and buffer was carefully removed using a pipette to a final volume of approximately 40 μl. A cell count was performed to assess viability before cells were subjected to library preparation. The scRNA-seq library was generated using the 10X Single Cell Chromium system as per the manufacturer’s guidelines at the Rockefeller University Genomics Core Facility. The library was then sequenced on an Illumina NovaSeq SP flowcell, ensuring a minimum sequencing depth of 30,000 reads per cell using read lengths of 26 bp for read 1, 8 bp for the i7 index, and 98 bp for read 2.

### Library preparation for bulk RNA-seq.

RNA was isolated using RNAClean XP beads (Agentcourt, A63987) on a magnetic stand (DynaMag, Invitrogen 12331D). Reverse transcription primers were: P1-RNA-TSO: Biot-rArArUrGrArUrArCrGrGrCrGrArCrCrArCrCrGrAr UrNrNrNrNrNrNrGrGrG; P1-T31: Biot-AATGATACGGCGACCACCGATCG31T; P1-PCR: Biot-GAATGATACGGCGACCACCGAT. RNA was eluted for 1 min at room temperature in cDNA synthesis mix 1 (0. 5 μl P1-T31 (20 μM), 0.3 μl Rnasin plus (Promega, N2615), 1.5 μl 10 mM dNTP, 3.5 μl 10 mM Tris pH 7.5, 0.5% IGEPAL CA-630 (Sigma) and 1.7 μl Rnase free ddH2O) and the beads were pipetted up and down ten times. The eluted sample was then incubated for 3 min at 72 °C, followed by 1 min on ice, then 7.5 μl of mix 2 was added (3 μl 5Å~ FS Buffer SS, 0.375 μl 100 mM DTT, 0.375 μl Rnasin plus, 0.5 μl P1-RNA-TSO (40 μM), 0.75 μl Maxima RT Minus H (Thermo Scientific, EP0751), 1.8 μl 5M Betaine (Sigma, B0300), 0.9 μl 50 mM MgCl2 and 0.175 μl Rnase free ddH2O. Reverse transcription occurred during a thermal cycle of one cycle (90 min at 42 °C), 10 cycles (2 min at 50 °C, 2 min at 42 °C) and one cycle (15 min at 70 °C), and the product was kept at 4 °C. The cDNA was then amplified using 15 μl of the reverse transcription product, 20 μl 2Å~ KAPA HiFi HS Ready Mix (Kapabiosystems, KK2601), 1.5 μl P1-PCR (10 μM), and 3.5 μl Rnase-free ddH2O. Amplification occurred during the following cycles: one cycle of 3 min at 98 °C, 20 cycles of (15 s at 98 °C, 20 s at 67 °C, 6 min at 72 °C), and one cycle of 5 min at 72 °C, and the product was kept at 4 °C. PCR product (20 μl) was cleaned up using 16 μl RNAClean XP beads. The cDNA was eluted in 20 μl Rnase-free ddH2O and kept at −20 °C. Isolated amplicons were confirmed to be 1,500–2,000 bp long by a High Sensitivity DNA Assay (Bioanalyzer). Concentration of all samples were measured on a Qubit fluorometer (Thermo Fisher), all samples were adjusted to 0.2 ng/μl with ddH2O, and 2.5 μl cDNA was subjected to Nextera XT DNA Library preparation (Illumina) using a Nextera XT Index Kit (Illumina, FC-131–1002) according to the manufacturer’s protocol, except that all volumes were used at 0.5x the indicated volumes. Sample quality was again verified by Bioanalyzer, and sample concentrations were measured on the Qubit fluorometer and adjusted to a concentration of 4.54 ng/μl. All samples were pooled at equal contributions and run in multiple lanes. Sequencing was performed using 75-base single-end reading on a NextSeq instrument (Illumina).

### Single-cell RNA-Seq analysis.

Fastq sequence files from smartseq2 generated from libraries were aligned to the mouse genome (mm39) associated with the mouse transcriptome annotations (v. gencode M29) using STAR (v. 2.7.10a; Dobin et al., 2013). Subsequently, genome-mapped BAM files were processed through RSEM (v. 1.3.1; Li and Dewey, 2011) for read quantification. Subsequently, the matrix of gene/UMI counts were used as input for analysis by the R package Seurat (v. 4.1.2.; Stuart et al., 2019). Next, the dataset was normalized by the LogNormalize method implemented by Seurat. We “regressed out” the mitochondrial genes and library size bias with the ScaleData function to control unwanted experimental noise sources. Additionally, cells containing more than 5% sequence reads aligned to mitochondrial genes were excluded before normalization. Single cells were clustered, and gene expression was evaluated with the Seurat workflow. Gene signature scoring was performed by using Seurat’s AddModuleScore function. Heatmaps were produced with the ComplexHeatmaps package for R (10.1093/bioinformatics/btw313). Differentially expressed gene analyses between clusters or biological groups were determined by the Seurat function FindAllMarkers with the non-parametric Wilcoxon Rank sum test, and *P*-values were adjusted using the Bonferroni correction. Genes were considered for downstream analysis when having a log2 fold change greater or smaller than 0.5 and exhibiting an adjusted *P*-value of at least 0.05.

### Bulk RNA-Seq Analysis.

Raw fastq files were aligned and quantified with STAR (v. 2.7.10a) by using the mouse genome (mm39) and the mouse transcriptome (gencode M29) (v. 2.7.10a; Dobin et al., 2013). Next, the count matrix was imported to the R environment and processed by the DESeq2 package (v. 1.34) pipeline (doi:10.1186/s13059–014- 0550–8). Briefly, genes from samples expressing less than ten reads were pre-filtered. Gene expression among samples was normalized by applying a negative binomial distribution model. The Wald test was employed to determine differential gene expression between conditions. Genes containing adjusted *P*-values (FDR) less than 0.1 were considered for downstream analysis, and log2 fold changes were shrunk using the apeglm algorithm.

### Adoptive T cell transfer.

naïve CD4^+^ T cells from spleen and lymph nodes were isolated by negative selection using biotinylated antibodies against CD8α, CD25, CD11c, CD11b, TER-119, NK1.1, and B220 and anti-biotin MACS beads (Miltenyi Biotec). The purity of transgenic CD4^+^ OT-II T cells was verified by flow cytometry (CD45.1^+^Vα2^+^Vβ5^+^CD25^−^, typically >90%). 1 × 10^6^ OT-II cells were transferred by retro-orbital injection under isoflurane gas anesthesia.

### Oral antigen administration.

OVA (grade III, Sigma, A5378) was administered intragastric at 50 mg in 200 μl PBS using plastic gavage needles. OVA was given 16–18 h after adoptive OT-II cell transfer. For the experiments where OVA was given in solid food, chow diet was supplemented with 1% of OVA, provided *ad libitum* 16–18 h after adoptive OT-II cell transfer.

### Bone marrow chimaeras.

C57BL/6 recipient mice were lethally irradiated with two doses of 450 Rads given 4 h apart. After irradiation, recipients were reconstituted by intravenous injection of hematopoietic cells collected from femurs and tibiae of donor mice, as described before (20). Mice were used for experiments 8–12 weeks after irradiation.

### *In vitro* dendritic cell-T cell co-culture.

LIPSTIC substrate was delivered *in vivo* and dendritic cells were collected from the duodenum-gLN, as described above. For experiments on Fig. 1f, g and Supplementary Fig. 3a, 150 Biotin^−^, Biotin^+^ or total DCs were sorted into U bottom 96well plate containing supplemented RPMI and 2% of T-stim media (VWR). 750 naïve CD4^+^ OT-II *Foxp3*^RFP^ CFSE-labeled or CTV-labeled T cells from the spleen were sorted into the corresponding wells. Cells were incubated at 37°C, 5% CO2 for 96 h. To determine the number of proliferated cells, all cells in each well were recorded by FACS and gated based on CFSE or CTV staining. For all other co-culture experiments, 1 μM of OT-II peptide (chicken OVA amino acids 323–339 - Anaspec AS-27024) or TN peptide (β-hex peptide sequence YKGSR–WLN - GenScript) (37) was added to the wells. 50 Biotin^−^ or Biotin^+^ DCs and 750 naïve CD4^+^ OT-II or TN CFSE-labeled or CTV-labeled T cells from the spleen were sorted into U bottom 96well plate containing supplemented RPMI and 2% of T-stim media. Cells were incubated at 37°C, 5% CO2 for 96 h, followed by surface and intracellular FACS staining, as described above. Freshly isolated congenic CD45.2^+^ splenocytes were added to the wells prior staining to prevent cell loss. Naïve CD4^+^ OT-II or TN T cells from the spleen were first isolated by negative selection and CFSE-labeled or CTV-labeled, as described above. These cells were then sorted as Aqua^−^CD45.1^+^TCRVα2^+^CD62L^+^CD44^−^CD25^−^. Dendritic cells were sorted as Aqua^−^ CD45.2^+^CD45.1^−^Lin^−^(TCRb^−^B220^−^CD64^−^) CD11c^hi^MHC-II^int/hi^Biotin^−^ or Biotin^+^. cDC1s were sorted as Aqua^−^ CD45.2^+^CD45.1^−^Lin^−^(TCRb^−^B220^−^CD64^−^) CD11c^hi^MHC-II^hi^CD103^+^CD11b^−^ and cDC2s were sorted as Aqua^−^ CD45.2^+^CD45.1^−^Lin^−^(TCRb^−^B220^−^CD64^−^)CD11c^hi^MHC-II^hi^CD103^+/−^CD11b^+^.

### *Strongyloides venezuelensis* passage and infection.

*S. venezuelensis* was maintained in our facility in NSG mice by subcutaneous infection with 1000 stage 3 (L3) larvae, resulting in chronic infection of this strain. For each experiment, feces of infected NSG mice were collected and spread on Whatman paper, which was placed into a beaker with water and incubated at 28C for 3 days. Mice were infected subcutaneously with 700 L3 larvae in 200 ml water per mouse. *S. venezuelensis* was passaged periodically by infecting naïve adult NSG mice.

### *Heligmosomoides polygyrus* infection.

*H. polygyrus* larvae was kindly provided by William Gause (Rutgers University). Mice were infected by oral gavage with 125 third-stage larvae of *H. polygyurs* in 100 μl of PBS. For the oral tolerance experiments on fig. S5, A to D, C57BL/6 mice were treated with two intragastric doses of Pyrantel Pamoate (300 μl of 10mg/ml in PBS) to clear *H. polygyurs* infection.

### OVA/cholera toxin allergy model.

Eight days after oral administration of OVA, 1 mg OVA + 20 μg cholera toxin (100B; List Biological) in 0.2 M sodium bicarbonate were provided by gavage once per week for 4 weeks, followed by a challenge 7 d after the final dose. Implantable electronic temperature probes (Avidity IPTT-300) were injected s.c. 1 d prior to the challenge. Mice were challenged with 5 mg OVA i.p., and body temperature was measured every 10 min for 50 min.

### Alum immunization and airway challenge.

Twelve days after oral administration of OVA, 4 μg of endotoxin-free OVA antigen adsorbed to 40 μl Imject Alum Adjuvant (Fisher Scientific) was injected intraperitoneally in a final volume of 400 μl made up with PBS. Immunization was repeated after 7 days. To induce airway inflammation, mice were anaesthetized and intranasally administered 10 μg of sterile OVA grade VI in 50 μl PBS (25 μl per nostril) on days 14, 17 and 21 after the first intraperitoneal immunization.

### Bronchoalveolar lavage (BAL) and infiltrate analysis by flow cytometry.

Mice were anaesthetized by intraperitoneal injection of 0.35 ml 2.5% avertin (Sigma), the trachea was cannulated and lungs were lavaged once with 0.5 ml and then with 1.0 ml PBS. Total BAL cells were counted after erythrocyte lysis and stained for FACS analysis. Lungs were perfused via the right ventricle with 10 ml saline to wash out residual blood. One lobe was digested in 400 U/ml collagenase D/RPMI and processed for FACS analysis. Eosinophils were determined as CD45^+^SSA^hi^MHC-II^−^CD11b^+^Ly6G^int^SiglecF^+^.

### Anti-OVA IgG1 ELISA.

Enzyme-linked immunosorbent assays (ELISAs) were performed as described previously (54).

### Anti-OVA IgE ELISA.

Enzyme-linked immunosorbent assays (ELISAs) were performed using ELISA Kit Legend MAX^™^ Mouse OVA Specific IgE ELISA Kit with Pre-coated Plates (BioLegend, 439807), according to manufacturer’s instructions.

### Tamoxifen treatment.

Tamoxifen (Sigma-Aldrich) was dissolved in corn oil (Sigma- Aldrich) and 10% ethanol, shaking at 37°C for 30 min–1 h. Two doses of Tamoxifen (5 mg/dose) were administered to i*Sell*^Tomato^ mice via oral gavage at 50 mg/ml, at days 4 and 6 post *S.v.* infection.

### EdU Treatment and Detection.

0.8 mg/ml EdU was given to mice in the drinking water from 5 to 7 days post-infection with *S.v*.. Detection was performed using the Click-iT™ Plus EdU Flow Cytometery Assay kit (Thermo Fisher Scientific, C10634), according to manufacturer’s instructions.

### Statistical analysis.

Statistical analyses were performed in GraphPad Prism 9.0 software. Error bars indicate S.E.M. Comparisons between two treatment conditions were analyzed using two-tailed unpaired Student’s t-test. Wilcoxon signed-rank test was used for RNA-seq data (indicated in figure legends). Multivariate data were analyzed by applying one-way ANOVA or two-way ANOVA and Tukey’s multiple comparison post hoc test. A *P*-value of less than 0.05 was considered significant.

## Supplementary Material

suppl material

Data S1

Data S2

Data S3

Data S4

Science checklist

## Data Availability

All data needed to evaluate the conclusions in the manuscript are present in the paper or Supplementary Materials. The mouse sequencing data are available through the Gene Expression Omnibus under accession GSE281286. All code used for analysis in this manuscript are available at (56).
