# Supplementary material for: Identification of antigen-presenting cell-T cell interactions driving immune responses to food
Source: Science. Author manuscript; Available in PMC 2025 Apr 23. (PMC12017586; doi:10.1126/science.ado5088)
Supplement: suppl material [file NIHMS2067875-supplement-suppl_material.pdf]

Science
AAAS

Supplementary Materials for

Identification of antigen-presenting cell-T cell interactions driving immune responses to food

Maria C.C. Canesso[1,2]*, Tiago B.R. Castro[1,2,]†, Sandra Nakandakari-Higa[2,]†, Ainsley Lockhart[1], Julia Luehr[1], Juliana Bortolatto[2], Roham Parsa[1], Daria Esterházy[3], Mengze Lyu[4], Tian-Tian Liu[5], Kenneth M. Murphy[5], Gregory F. Sonnenberg[4], Bernardo S. Reis[1], Gabriel D. Victora[2,6,]*, Daniel Mucida[1,6,]*

Correspondence to: Maria C. C. Canesso, mcamposcan@rockefeller.edu; Gabriel D. Victora, victora@rockefeller.edu; Daniel Mucida, mucida@rockefeller.edu.

Science 387, eado5088 (2025)
DOI: 10.1126/science.ado5088

This PDF file includes:

Figs. S1 to S9
Tables S1

Other Supplementary Materials for this manuscript include the following:

MDAR Reproducibility Checklist
Data S1 to S4

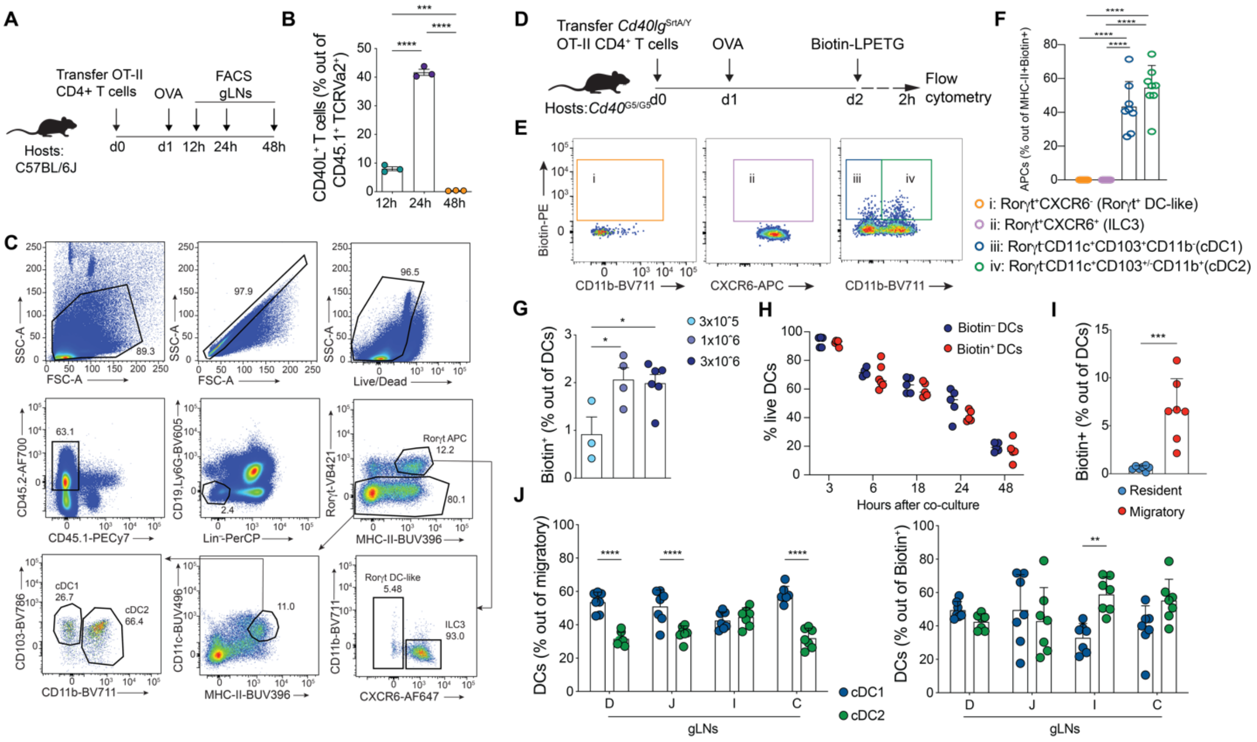

Fig. S1. Intestine-adapted LIPSTIC optimization. (A, B) CD45.2 C57BL/6 mice were adoptively transferred with 1 x 10^6 naive CD45.1 $CD4^+$ OT-II T cells prior to one dose of intragastric OVA. Analyses were carried out at indicated time points. **(A)** Experimental setup for panel (B). **(B)** Percentage of $CD40L^+$ T cells among $CD45.1^+TCRV\alpha2^+$ cells in D-gLNs (n = 3 mice per group). **(C)** Flow plots showing the general gating strategy for APCs. **(D)** Experimental setup for panel *E and F*. **(E and F)** CD45.2 *Cd40*$^{G5/G5}$ mice were adoptively transferred with 1 x 10^6 naive CD45.1 $CD4^+$ *Cd40lg*$^{SrtA/Y}$ OT-II T cells prior to one dose of intragastric OVA. Cell-cell interaction was revealed by LIPSTIC protocol 24 h later. **(E)** Representative flow plots showing percentage of labeled APCs in the D-gLNs and **(F)** quantification of data. Each dot represents one mouse (n = 4 mice per group, pool of two independent experiments). **(G)** CD45.2 *Cd40*$^{G5/G5}$ mice were adoptively transferred with 3 x 10^5, 1 x 10^6 or 3 x 10^6 of naive CD45.1 $CD4^+$ *Cd40lg*$^{SrtA/Y}$ OT-II T cells prior to one dose of intragastric OVA. Cell-cell interaction was revealed by LIPSTIC protocol 24 h later. Percentage of labeled DCs in the D-gLNs (n = 3 to 6 mice per group). **(H)** Sorted D-gLNs $biotin^+$ or $biotin^-$ DCs were co-cultured in vitro with naïve $CD4^+$ OT-II CTV-labeled T cells for 3, 6, 18, 24 or 48 h prior analysis. Graph shows the percentage of live DCs at indicated time points. Each dot represents one mouse (n = 5 per group). **(I and J)** CD45.2 *Cd40*$^{G5/G5}$ mice that were adoptively transferred with 1 x 10^6 naive CD45.1 $CD4^+$ *Cd40lg*$^{SrtA/Y}$ OT-II T cells. Mice received 1% of OVA diet for 24 h prior to LIPSTIC protocol revealing cell-cell interaction. **(I)** Percentage of labeled resident and migratory DCs in D-gLNs. **(J)** Percentage of cDC1 and cDC2 out of total migratory DCs (left) and percentage of cDC1 and cDC2 out of $biotin^+$ DCs (right). Each dot represents one mouse (n = 3, 4 mice per group, pool of two independent experiments). D, duodenum; J, jejunum; I, ileum; C, colon. In graphs, the height of bars indicate mean, and error bars indicate SD. *P*-values were calculated by one-way ANOVA (B) and (G), two-way ANOVA (F) and (J), or unpaired *t*-test (H) and (I). Statistical significance denoted as $^*P < 0.05$, $^{**}P < 0.01$, $^{***}P < 0.001$, $^{****}P < 0.0001$; only p values < 0.05 are shown.

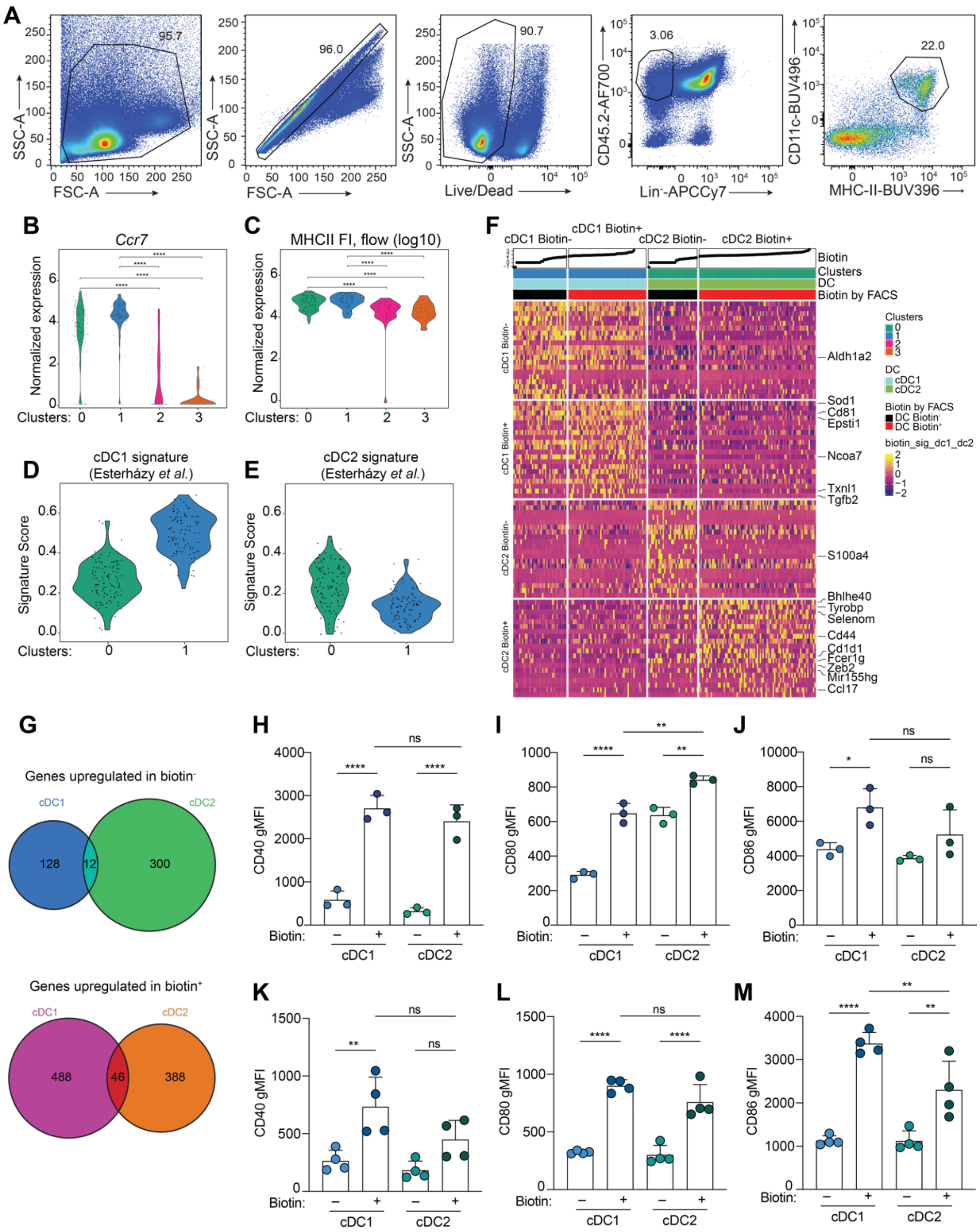

Fig. S2. Single-cell transcriptomics of D-gLNs DCs. CD45.2 *Cd40*$^{G5/G5}$ mice were adoptively transferred with 1 x 10^6 naive CD45.1 $CD4^+$ *Cd40lg*$^{SrtA/Y}$ OT-II T cells prior to one dose of intragastric OVA. Cell-cell interaction was revealed by LIPSTIC protocol 24 h later. D-gLNs biotin⁻ and biotin⁺ DCs were single-cell sorted and subjected to scRNA-seq. **(A)** Flow plots showing the gating strategy for sorting. **(B)** *Ccr7* expression in different transcriptional clusters as defined in Fig 2A. **(C)** Fluorescence intensity (FI) of MHC-II staining in transcriptional clusters as defined in Fig 2A. FI data was obtained from flow cytometry index-sorting files. Expression of **(D)** cDC1 and **(E)** cDC2 gene expression signatures, obtained from the literature (*3*), in transcriptional Clusters 0 and 1. **(F)** Heatmap showing expression of genes significantly modulated in biotin⁻ and biotin⁺ cDC1 and cDC2. The uppermost rows show biotin FI from flow cytometry,

followed by transcriptional clusters as shown in Fig. 2A and cDC1 or cDC2 as defined in (d and e). Selected genes are indicated in the right. **(G)** Venn diagram of differentially transcribed genes between biotin$^-$ and biotin$^+$ cDC1 and cDC2. **(H)** CD40, **(I)** CD80 and **(J)** CD86 geometric mean fluorescence intensity (gMFI) of biotin$^-$ and biotin$^+$ cDC1s and cDC2s from D-gLNs (n = 3 mice per group). **(K to M)** CD45.2 *Cd40*$^{G5/G5}$ mice that were adoptively transferred with 1 x 10^6 naive CD45.1 CD4$^+$ *Cd40lg*$^{SrtA/Y}$ OT-II T cells. Mice received 1% of OVA diet for 24 h prior to LIPSTIC protocol revealing cell-cell interaction. **(K)** CD40, **(L)** CD80 and **(M)** CD86 geometric mean fluorescence intensity (gMFI) of biotin$^-$ and biotin$^+$ cDC1s and cDC2s from D-gLNs (n = 4 mice per group). In graphs, the height of bars indicate mean, and error bars indicate SD. *P*-values were calculated by Wilcoxon signed-rank test in (B), (C), (D) and (E) or one-way ANOVA in (H), (I), (J), (K), (L) and (M). Statistical significance denoted as not significant (ns), *P < 0.05, **P < 0.01, ****P < 0.0001.

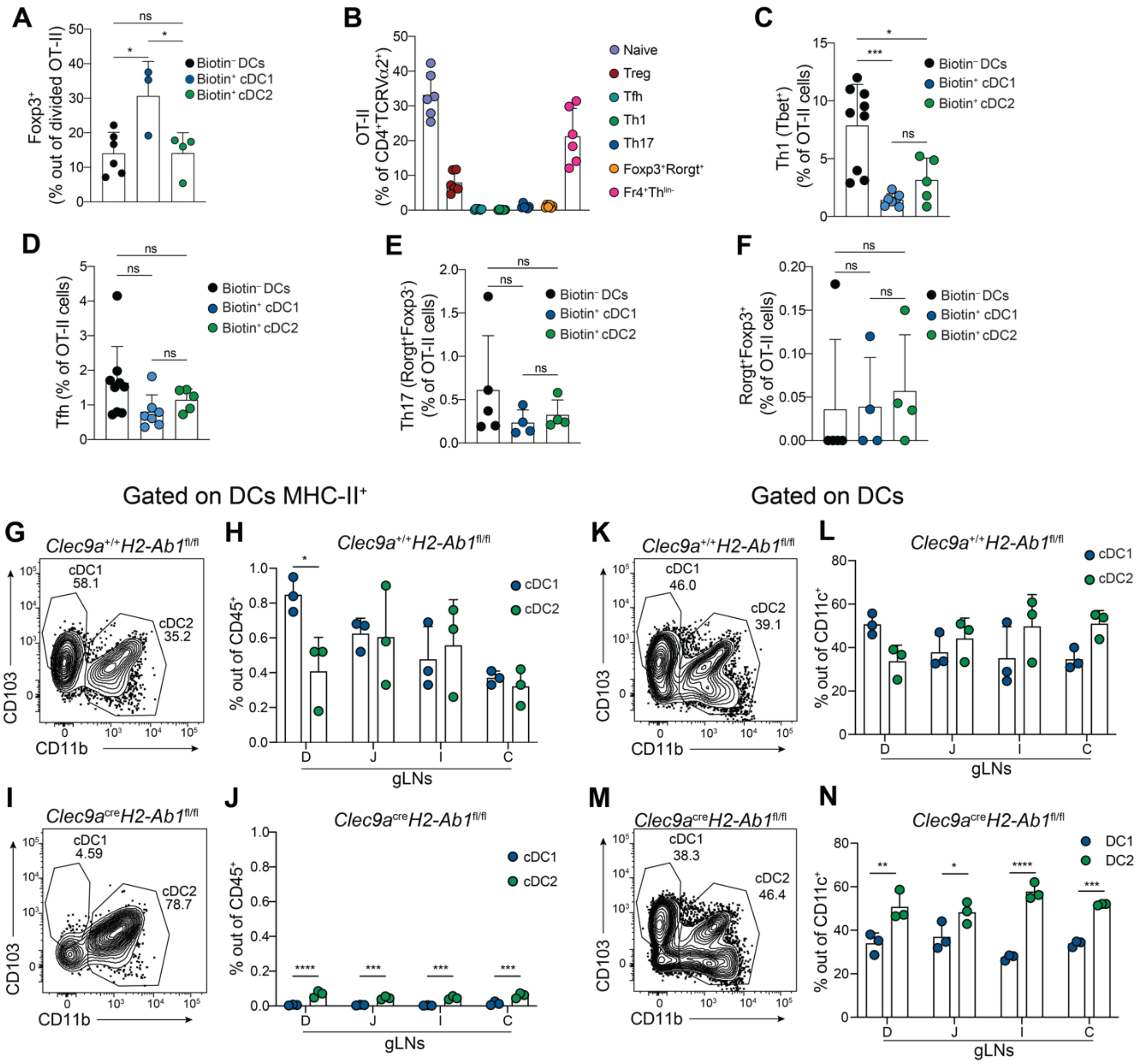

Fig. S3. Food-specific T cell response induced by cDCs subsets (A) Naïve OT-II CTV-labeled T cells were co-cultured *in vitro* with sorted D-gLNs biotin⁻ DCs, biotin⁺ cDC1s or biotin⁺ cDC2s for 96 h. Percentage of Foxp3⁺ cells among proliferated OT-II T cells. Each dot represents one mouse (n = 3, 4 mice per group, pool of two independent experiments). **(B)** CD45.2 C56BL/6 mice were adoptively transferred with 1 x 10^6 naive CD45.1 CD4⁺ OT-II T cells. Mice received two doses of intragastric OVA 48 h and 24 h prior analysis. Percentage of naïve (CD44⁻), Tregs (Foxp3⁺), Tfh (CXCR5⁺), Th1 (Tbet⁺), Th17 (Rorγt⁺), Foxp3⁺Rorγt⁺ and Fr4⁺Th^{lin-} cells among CD4⁺TCRVα2⁺ (OT-II) T cells in D-gLNs. (n = 3 mice per group, pool of two independent experiments). Naïve OT-II CTV-labeled T cells were co-cultured *in vitro* with sorted D-gLNs biotin⁻ DCs, biotin⁺ cDC1s or biotin⁺ cDC2s in the presence of exogenous OT-II peptide for 96 h. Percentage of **(C)** Th1, **(D)** Tfh, **(E)** Th17 and **(F)** Foxp3⁺Rorγt⁺ cells among proliferated OT-II T cells. **(G to N)** CD45.2 *Clec9a*$^{+/+}$*H2-Ab1*$^{fl/fl}$ or *Clec9a*$^{+/cre}$*H2-Ab1*$^{fl/fl}$ mice were adoptively transferred with 1 x 10^6 naive CD45.1 CD4⁺ OT-II T cells. Mice received two doses of intragastric OVA 48 h and 24 h prior analysis. **(G and I)** Representative flow plots of D-gLNs showing percentage of cDC1 and cDC2 out of CD45.2 migratory DCs MHC-II^{hi} and **(H and J)** quantification of data. **(K and M)** Representative flow plots of D-gLNs showing percentage of cDC1 and cDC2 out of DCs (CD11c⁺) and **(L and N)** quantification of data. (n = 3 mice per group,

representative of two independent experiments). D, duodenum; J, jejunum; I, ileum; C, colon. In graphs, the height of bars indicate mean, and error bars indicate SD. *P*-values were calculated by one-way ANOVA in (A), (C), (D), (E) and (F), or two-way ANOVA in (H), (J), (L) and (N). Statistical significance denoted as not significant (ns), *P < 0.05, **P < 0.01, ***P < 0.001, ****P < 0.0001.

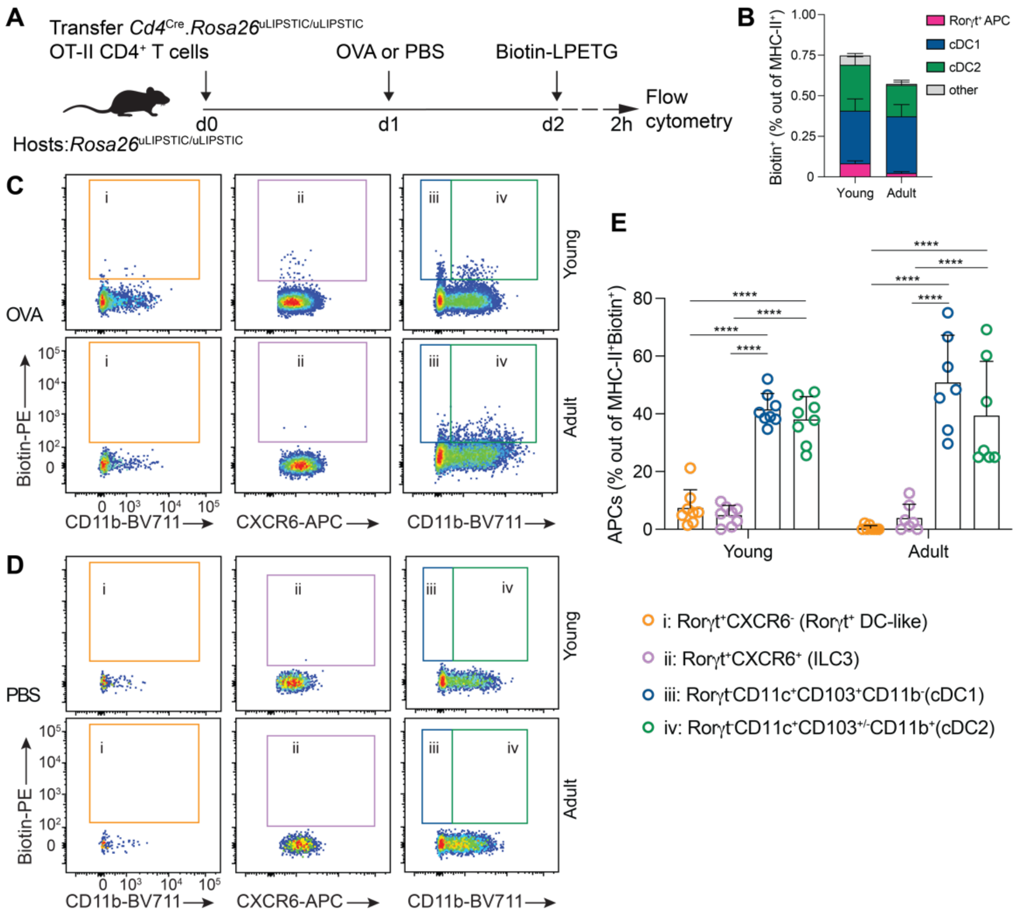

Fig. S4. Dietary-antigen presentation in young and adult mice. (A) Experimental setup for panel *B to E*. **(B to E)** CD45.2 *Rosa26*$^{uLIPSTIC/uLIPSTIC}$ mice were adoptively transferred with 3 x 10^6 naive CD45.1 *Cd4*^{Cre}.*Rosa26*$^{uLIPSTIC/uLIPSTIC}$ OT-II T cells prior to one dose of intragastric OVA or PBS. Cell-cell interaction was revealed by LIPSTIC protocol 24 h later. **(B)** Percentage of Biotin$^+$ cells among MHC-II$^+$ cells. Representative flow plots showing percentage of labeled APCs in the duodenal gLNs after intragastric OVA **(C)** or PBS **(D). (E)** Quantification of data as in (C). Each dot represents one mouse (n = 4 mice per group, pool of two independent experiments). In graphs, the height of bars indicate mean, and error bars indicate SEM (B) and SD (E). *P*-values were calculated by two-way ANOVA in (E). Statistical significance denoted as ****$P < 0.0001$; only p values < 0.05 are shown.

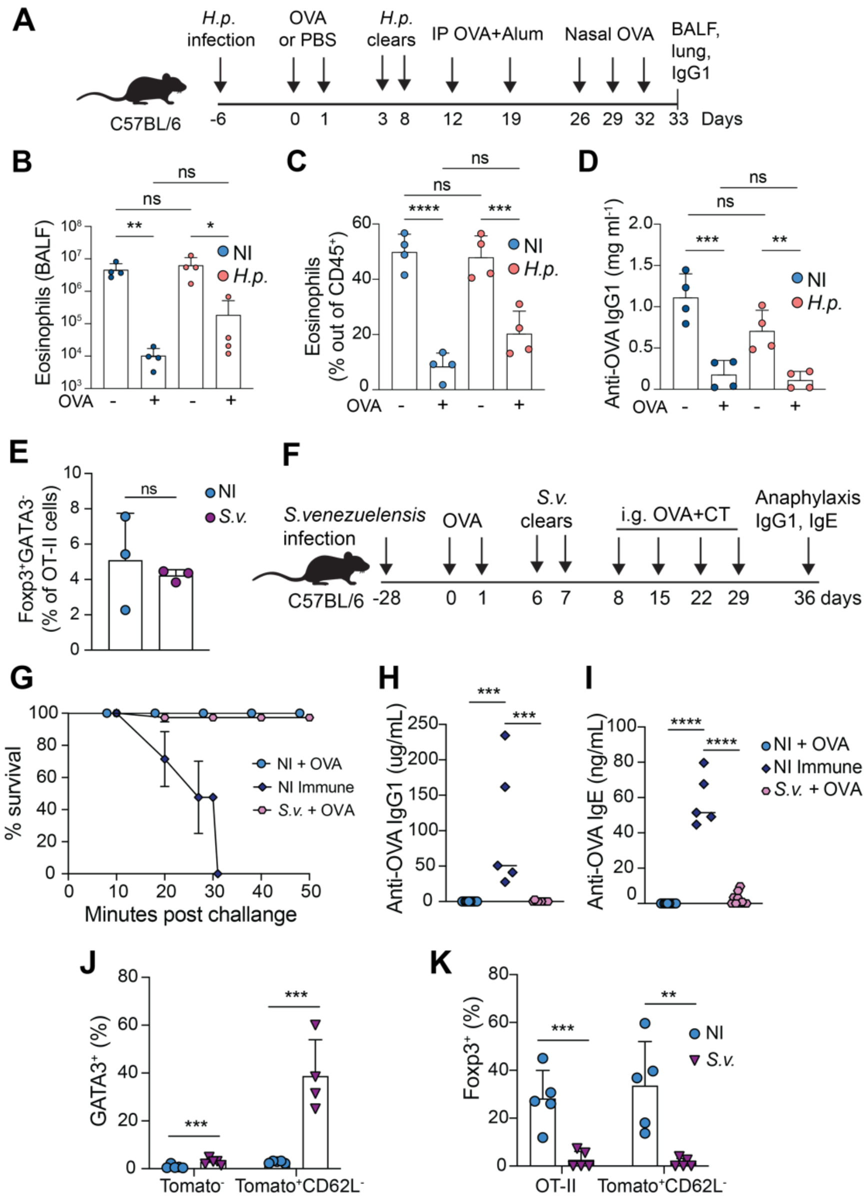

Fig. S5. Helminth infection impact in oral tolerance. **(A to D)** CD45.2 C57BL/6 mice were infected with *H. polygyrus (H.p.)* or not during antigen feeding (+OVA groups), or no feeding (–OVA groups), 18 days before first immunization with OVA–alum. **(A)** Scheme of oral tolerance experimental set up in *H.p.*-infected mice. **(B)** Total eosinophils in BALF, **(C)** Percentage of eosinophils among CD45$^+$ cells in lung tissue and **(D)** OVA-specific IgG1 levels in serum (n = 4 mice per group, representative of two independent experiments). **(E)** CD45.2 C57BL/6 mice were infected with *S. venezuelensis (S.v.)* 28 days prior adoptively transfer of 1 x 10^6 naive CD45.1 CD4$^+$ OT-II T cells. Mice received two doses of intragastric OVA 48 h and 24 h prior analysis. Non-infected mice (NI) were used as control. Percentage of Foxp3$^+$ cells among CD45.1 TCRVα2$^+$ (OT-II) T cells in D-gLNs. (n = 3 mice per group, representative of two independent experiments). **(F)** Experimental setup for panel (G to I). **(G)** Anaphylaxis as measured by survival of mice at the indicated times after intraperitoneal OVA injection (challenge), following four weekly doses of OVA+cholera toxin (CT). **(H)** OVA-specific IgG1 or **(I)** OVA-specific IgE levels in serum as measured by ELISA (n = 3, 5 mice per group, pool of two independent experiments).

(J and K) CD45.2 i*Sell*^{Tomato} mice were infected with *S.v.* 5 days prior adoptively transfer of 1 x 10^6 naive CD45.1 $CD4^+$ OT-II T cells. Mice received two doses of tamoxifen, at days 4 and 6 post infection, and two doses of intragastric OVA at days 6 and 7 post infection. Analyses were carried out at day 13 post infection. **(J)** Percentage of $GATA3^+$ out of $Tomato^-$ or $Tomato^+CD62L^-$ T cells. **(K)** Percentage of $Foxp3^+$ out of OT-II or $Tomato^+CD62L^-$ T cells. (n = 2, 3 mice per group, pool of two independent experiments). In graphs, the height of bars indicate mean, and error bars indicate SD. *P*-values were calculated by two-way ANOVA in (B), (C), (D), (J) and (K), by unpaired *t*-test in (E), or one-way ANOVA in (H) and (I). Statistical significance denoted as not significant (ns), *$P < 0.05$, **$P < 0.01$, ***$P < 0.001$, ****$P < 0.0001$.

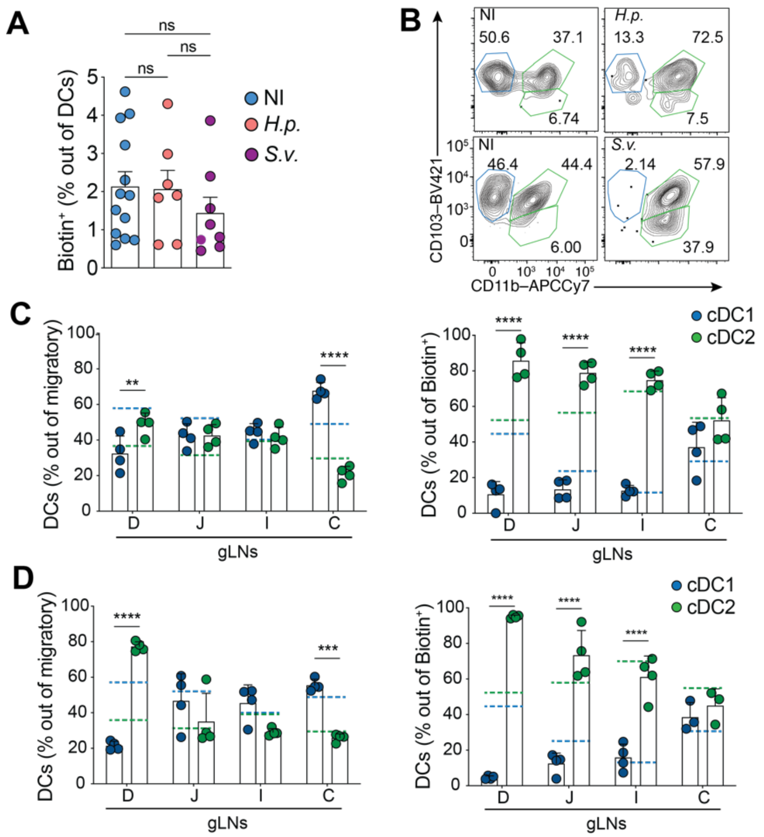

Fig. S6. Frequencies of cDC subsets in gLNs during *Strongyloides venezuelensis* or *Heligmosomoides polygyrus* infection. (A and B) CD45.2 *Cd40*$^{G5/G5}$ mice were infected with *S. venezuelensis* (*S.v.)* or *H. polygyrus* (*H.p.)* 5 days prior adoptively transfer of 1 x 10^6 naive CD45.1 CD4$^+$ *Cd40lg*$^{SrtA/Y}$ OT-II T cells. Animals received 1 dose of intragastric OVA and cell-cell interaction was revealed by LIPSTIC protocol 24 h later. Non-infected mice (NI) were used as control. **(A)** Percentage of labeled DCs in the D-gLNs of NI, *H.p.*- or *S.v*-infected mice (n = 2, 4 mice per group, pool of three independent experiments). **(B)** Representative flow plots showing percentage of biotin$^+$ cDC1 (blue) and cDC2 (green) in D-gLNs of *H.p* or *S.v* infected mice. Quantification of data for **(C)** *H.p.*-infected mice and **(D)** *S.v*-infected mice. Dashed lines show mean value of cDC1 (blue) or cDC2 (green) percentage at steady state (NI). D, duodenum; J, jejunum; I, ileum; C, colon. In graphs, the height of bars indicate mean, and error bars indicate SD. *P*-values were calculated by one-way ANOVA in (A) or two-way ANOVA in (C) and (D). Statistical significance denoted as not significant (ns), $^{**}P < 0.01$, $^{***}P < 0.001$, $^{****}P < 0.0001$.

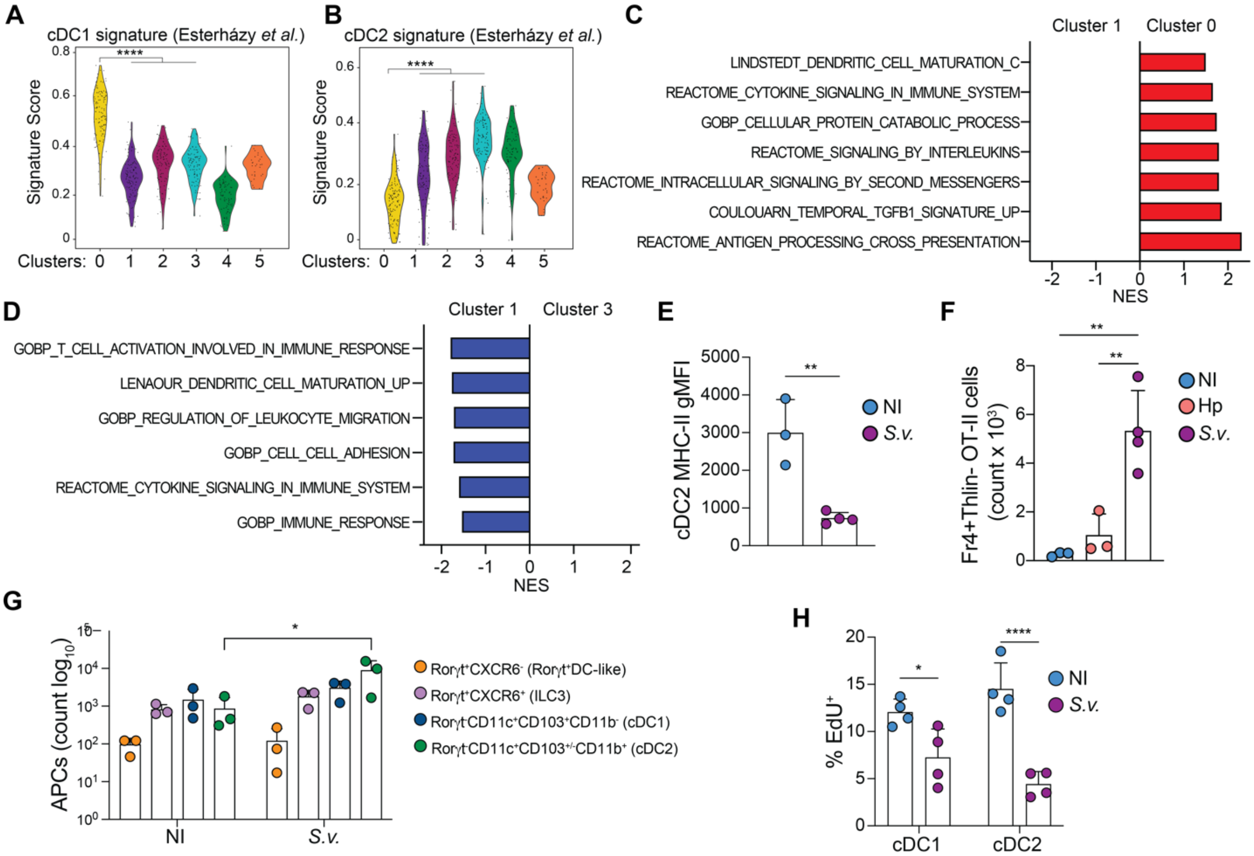

Fig. S7. Transcriptomics of D-gLNs DCs from *Heligmosomoides polygyrus* or *Strongyloides venezuelensis* infected mice. Expression of **(A)** cDC1 and **(B)** cDC2 gene expression signatures, obtained from the literature (*3*), in transcriptional clusters as defined in Fig. 4A. Differentially expressed gene signatures of D-gLNs DCs between **(C)** Cluster 0 *vs* Cluster 1 or **(D)** Cluster 1 *vs* Cluster 3. **(E)** MHC-II geometric mean fluorescence intensity (gMFI) of cDC2s from D-gLNs of non-infected (NI) or *S.v.*-infected mice (n = 3, 4 mice per group). **(F and G)** CD45.2 C56BL/6 mice were infected with *S.v.* or *H.p.* 5 days prior adoptively transfer of 1 x 10^6 naive CD45.1 $CD4^+$ OT-II T cells. Mice received two doses of intragastric OVA 48 h and 24 h prior analysis. **(F)** Number of $Fr4^+Th^{lin-}$ OT-II T cells and **(G)** APCs in D-gLNs. (n = 3 mice per group, representative of two independent experiments). **(H)** C57BL/6 mice received EdU in the drinking water at days 5-7 post-infection with *S.v.* Percentage of EdU^+ cDC1 and cDC2 in D-gLNs of non-infected (NI) and *S.v.* infected mice. In graphs, the height of bars indicate mean, and error bars indicate SD. *P*-values were calculated by Wilcoxon signed-rank test in (A) and (B), by unpaired *t*-test in (E), one-way ANOVA in (F) and two-way ANOVA in (G) and (H). Enrichment *P*-values were calculated by the fgsea package for R and corrected with the FDR (C) and (D). Statistical significance denoted as *P < 0.05, **P < 0.01, ****P < 0.0001; only p values < 0.05 are shown.

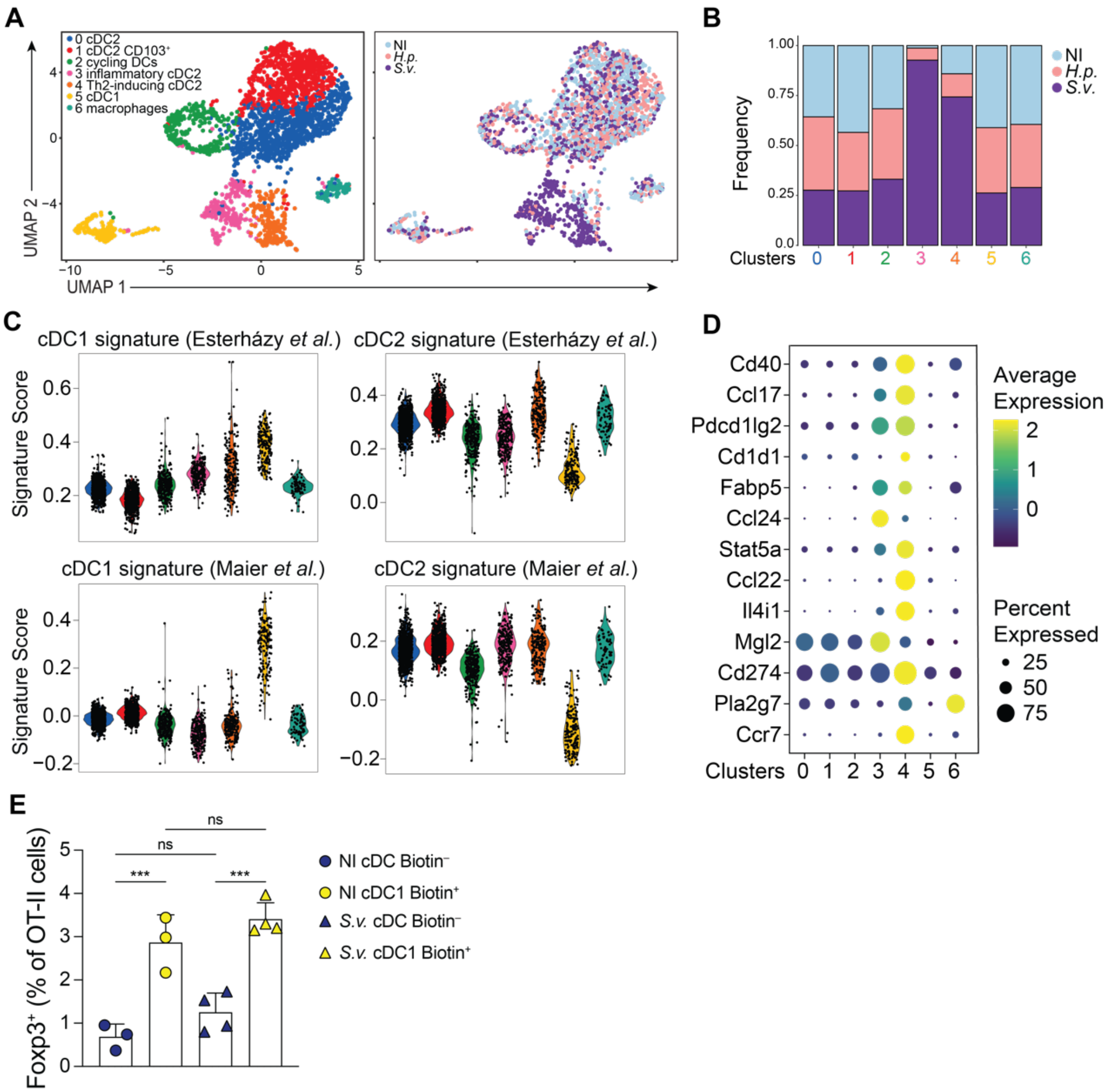

Fig. S8. Profile of cDCs in the duodenum lamina propria of helminth infected mice. (A-D) C57BL/6 mice were infected with *Sc.*B. or *H.p*. Non-infected mice (NI) were used as control. **(A)** UMAP plot showing clustering of DCs sorted from the duodenum LP of NI mice or mice infected with *H.p* or *S.v*. (2567 cells were analyzed). Cells were pooled from 3-4 mice. **(B)** Proportion of cells in each transcriptional cluster among NI, *H.p* or *S.v.* infected mice. **(C)** Expression of cDC1 and cDC2 gene expression signatures, obtained from the literature (*3, 55*), in transcriptional clusters as defined in (A). **(D)** Dot plot showing expression of genes differentially expressed between Clusters. **(E)** Percentage of Foxp3$^+$ cells among proliferated OT-II CFSE-labeled T cells *in vitro* after 96 h of co-culture with D-gLN NI or *S.v* biotin$^-$ cDC or biotin$^+$ cDC1. Each dot represents a pool of mice (n = 3 to 6 mice per group, representative of two independent experiments). Height of bars indicate mean, and error bars indicate SD (G). *P*-values were calculated using Wilcoxon signed-rank test or two-way ANOVA in (E). Statistical significance denoted as not significant (ns), ***P < 0.001.

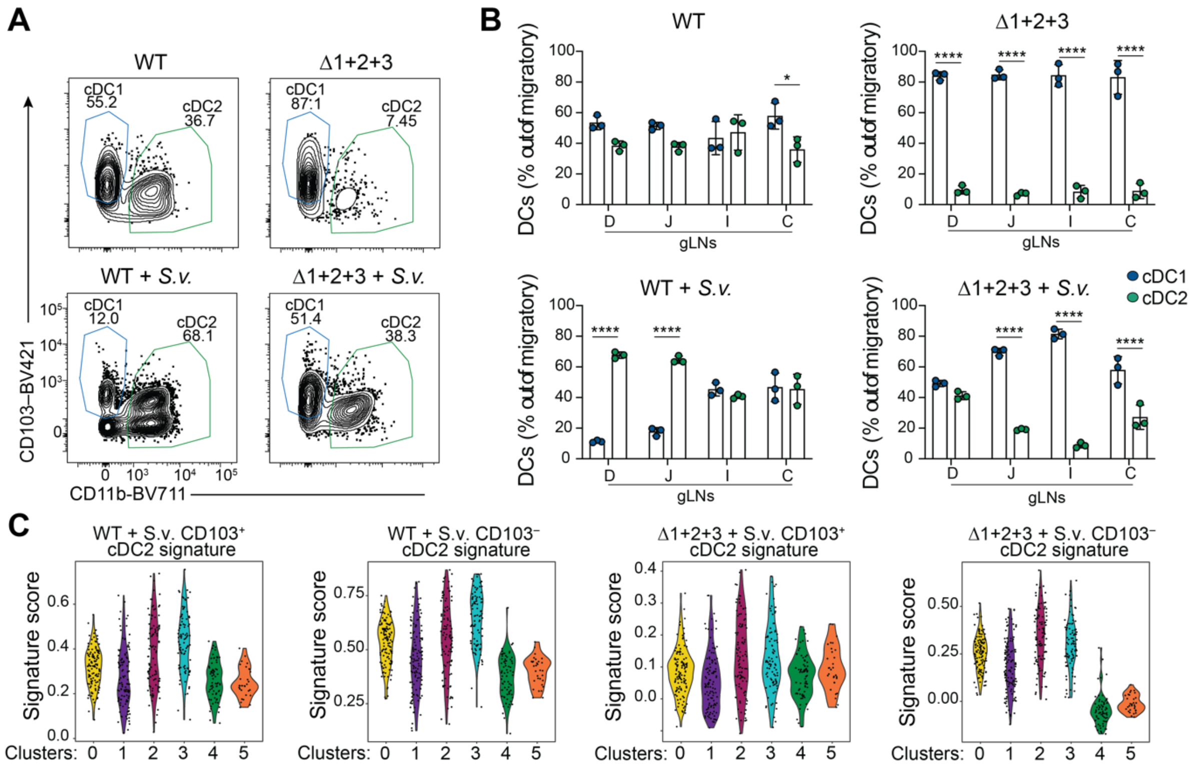

Fig. S9. cDCs profile in gLNs of WT and Δ1+2+3 chimera mice infected with *Strongyloides venezuelensis*. Bone marrow chimera (BMC) mice reconstituted with C57BL/6 (WT) or Δ1+2+3 cells. Mice were infected with *S.v.* as in Fig. 4F. **(A)** Representative flow plots showing percentage of cDC1 (blue) and cDC2 (green) in D-gLNs of non-infected or *S.v*-infected WT and Δ1+2+3 chimera mice. **(B)** Quantification of the data. Each dot represents one mouse (n = 3 mice per group, representative of two independent experiments). **(C)** Expression of D-gLNs (CD11b+CD103± "cDC2s") signatures, as indicated, in transcriptional clusters as defined in Fig. 4a. Transcriptional profiling of these cDC2s revealed that they express the signatures of Clusters 2 and 3 from the D-gLNs. D, duodenum; J, jejunum; I, ileum; C, colon. In graphs, the height of bars indicate mean, and error bars indicate SD. *P*-values were calculated two-way ANOVA in (B) or using Wilcoxon signed-rank test in (C). Statistical significance denoted as *P < 0.05, ****P < 0.001.

Table S1: Top 50 differentially expressed genes as in Fig. 2.

**Genes upregulated in Biotin- DCs**			**Genes upregulated in Biotin+ DCs**		
DC1	DC2	Shared between DC1 and DC2	DC1	DC2	Shared between DC1 and DC2
Slc26a2	Ubald2	Gm28900	Cox6a1	Gm37376	Ebi3
Mir703	Ptpmt1	Dcaf10	Dpysl2	Gramd1b	Supt6
Acot10	H3f3a	Gm10257	Ssr2	Mtdh	Rhof
Zc3h3	Rps5	Rasa4	Birc2	Strbp	Exoc4
Papss2	Cmip	6430531B16Rik	Aup1	Ywhag	Cd86
Gtf2f2	Mcph1	H2-Eb1	Procr	Gm12216	Cd40
Src	Ptchd1	H2-Aa	Sft2d1	Fuca1	Dock10
Gm43712	Ap3s1-ps2	Snrpd3	Gm16712	Cbx3	AI413582
Dmtn	Stip1	Cd74	Gm12166	Selenom	Zfp263
Rgs2	Atox1	Hspd1-ps3	Mbnl1	Atp5k	Pdia6
Gm10045	Gmeb1	Rps15	Plscr1	Csnk1d	Iqsec1
Tm4sf5	Arntl	Cytip	H2-M3	Bnip2	Ccl22
Trip4	Arpc2		Srsf9	Dscaml1	Ptdss2
Cs	Capns1		Ankrd17	Gbp4	Gm43305
Rexo2	Adam11		Itgav	Rnf115	Psd3
Rps7	Resf1		Glrx	Gal3st2	Phb
Gm7353	Phf11a		Pirb	Msn	Snrk
Gm10080	Cst3		Rnf167	Aco1	Snx17
Ufc1	Gm2395		Bud23	Arfrp1	Nfatc1
Mast2	Rpl30-ps10		Camk2g	Vta1	Wasf2
Kif3c	Rps18		Snw1	Tmem160	Ccrl2
Rpl10-ps3	Gstt1		Paip1	Edem1	Lacc1
Gm11793	Shroom1		Sepsecs	Chst15	Pdcd4
Pdzph1	Snhg16		Cfdp1	Dusp22	Dguok
Lrrc28	Car9		Nfkb1	Ccl17	Arhgap17
Terf2	Sugp2		Pik3c3	Iqgap1	Klhl22
Plppr3	Ndrg4		Ubtf	Zfp706	Shprh
Prmt9	Gm7367		Mkrn1	Rtcb	Pbxip1
Gm15596	Frg2f1		Ddx41	Abcg1	Cd82
Gm4575	Gm16199		1810026B05 Rik	Ikbkb	Htatsf1
Gm43462	Gm21119		Tnfrsf14	H2-K1	Clstn1
C230096K16 Rik	Gm21887		Gsk3b	Scyl1	Ppp4r3a
Gm44346	Gm5787		Rbm4b	Slx4ip	Bcl2l1
Rps18-ps4	Gm28052		Pum2	Inpp5j	Mier1
Rpl10	Gm47738		Pglyrp1	Slc25a39	Serpinb9b
Rap2b	Gm4600		Nip7	Casp4	Lcor
Prdx6	Rps2-ps7		Tgfb2	Stk24	Tmem209

As3mt	Tmem176a		Fez2	Gm40309	Fcho1
Hmbox1	Ptafr		Gm7666	Derl1	Nek6
Gm44694	Gm26631		Pim1	Trpc4ap	Rpn2
Hspa1a	Gm10293		Foxn3	Smarca2	Trappc6b
Eps15	Ddx39		Necap2	Il4ra	Zfp472
Rbm5	Gm37712		Oaz2	Smarca4	Ccnl2
9530078K11 Rik	Piezo1		Ier2	Emc10	Mtmr2
Nudt14	Tbcc		Mtch2	Gnpda1	Cdkn1a
Tlr9	Rbak		Tmem109	Ndfip1	Itsn2
Txndc17	Gm6421		Mrpl10	Mfsd1	
Castor2	Il2		Haghl	Sbno2	
Nudcd3	Osm		Golt1b	Ly75	
Mrpl19	Gm6192		Btla	Ttc9c	

Data S1. (separate file)

Antibodies used for flow cytometry

Data S2. (separate file)

Genes expressed in each cluster - Fig. 2

Data S3. (separate file)

Genes expressed in each cluster - Fig. 4

Data S4. (separate file)

Genes expressed in each cluster - Fig. S8
